# Effect of bispecific recombinant oncolytic adenovirus carrying apoptin on apoptosis of MCF-7 cells

**DOI:** 10.3389/fimmu.2025.1530583

**Published:** 2025-05-16

**Authors:** Shuang Chen, Wenyao Li, Xunzhe Yin, Cheng Hu, Yiquan Li, Xiaotong Shao, Xiao Li, Ningyi Jin

**Affiliations:** ^1^ School of Laboratory Medicine, Jilin Medical University, Jilin, China; ^2^ Institute of Military Veterinary Medicine, Academy of Military Medical Science, Changchun, China; ^3^ College of Medicine, Dalian University, Dalian, China; ^4^ Changchun Institute of Applied Chemistry, Chinese Academy of Sciences, Changchun, China

**Keywords:** MCF-7 cells, apoptin, proteomics, apoptosis, oncolytic adenovirus

## Abstract

**Objective:**

In this study, breast cancer cell line MCF-7 was infected with recombinant oncolytic adenovirus Ad-VT expressing apopsin protein, and its anti-tumour pathway was detected to determine its possible anti-tumour signalling pathway.

**Method:**

In this study, the inhibitory effect of recombinant oncolytic adenovirus Ad-VT on breast cancer cells was investigated through cell activity experiment and establishment of tumour bearing model in mice. Subsequently, in order to determine the apoptosis-inducing effect of recombinant oncolytic adenovirus on breast cancer cells, the effects of three recombinant oncolytic adenovirus on the apoptosis-inducing level of breast cancer cells were further analysed by Annexin V-FITC/PI detection, Hoechst staining, JC-1 staining and transmission electron microscopy. Then the differentially expressed proteins associated with apoptosis and possible signalling pathways were identified by proteomics and WB experiments.

**Results:**

*In vivo* and *in vitro* experiments showed that recombinant oncolytic adenovirus Ad-VT expressing apoptosis protein could induce apoptosis and inhibit the growth of MCF-7 cells. Proteomic analysis showed that differential genes were enriched in mTOR, MAPK and other pathways after Ad-VT infection of breast cancer cells, and the expression of S6K genes related to mTOR pathway was significantly increased in differential gene analysis, subsequently, the high expression of phosphorylated mTOR and S6K proteins was also determined by WB experiment, suggesting that Ad-VT may regulate the apoptosis of breast cancer cells through mTOR/S6K signalling.

**Conclusion:**

Ad-VT can significantly increase the apoptosis level of breast cancer cells, which may be induced by the mTOR/S6K signalling pathway. The results of this study provide a theoretical basis for the development of anti-tumour drugs based on Ad-VT in the future.

## Introduction

According to statistics from the World Health Organization, breast cancer is the most common cancer in the world and has the highest incidence rate among all cancers in women (1). Despite the continuous progress in the treatment of breast cancer, metastatic breast cancer is still incurable, and the overall 5-year survival rate is still less than 25% (2). An effective treatment strategy is urgently needed. Traditional treatments, such as radiotherapy, chemotherapy or surgery, exert direct inhibitory effects on tumours, but most of these treatments have side effects and cause significant damage to normal human tissues (3). Moreover, improvements in the survival rate and quality of life of most cancer patients are limited. Therefore, a treatment that has few side effects and can inhibit the metastasis of breast cancer must be developed.

Apoptosis is a gene-regulated cell suicide process mediated by the activation of caspases 3, 6 and 7. Apoptosis is a basic biological phenomenon of cells that plays a necessary role in the removal of unnecessary or abnormal cells in multicellular organisms (4–6). With the development of molecular biology technology, the process of apoptosis has become well understood, but the exact mechanism of apoptosis is not completely clear. The target of rapamycin (mTOR) signalling pathway plays an important role in tumorigenesis and development; thus, it has received an increasing amount of attention. mTOR is an atypical serine/threonine protein kinase belonging to the phosphatidylinositol 3-kinase-associated kinase (PIKK) family. In mammals, there are two main target molecules downstream of mTOR: ribosome S6 kinase (S6K) and eukaryotic translation initiation factor (eIF4E)-binding protein 1 (4E-BP1) (7, 8). The activation of mTOR leads to the phosphorylation of S6K, which induces apoptosis and inhibits tumour growth. However, there are few reports on whether Apoptin can induce the apoptosis of tumour cells by affecting the mTOR/S6K signalling pathway. In this study, proteomic sequencing was used to explore whether Ad-VT affects changes in the mTOR/S6K signalling pathway and induces the apoptosis of breast cancer cells.

Apoptin was one of the first tumour-selective anticancer genes isolated. Apoptin is derived from chicken anaemia virus (CAV) (9–11), which is a single-stranded DNA virus and is considered an apoptosis-inducing protein. The CAV genome contains three partially overlapping open reading frames that encode viral proteins from a single polycistronic mRNA: VP1, VP2 and VP3. The expression of VP3 alone is sufficient to trigger the death of chicken lymphoid T cells and myeloid cells but does not affect chicken fibroblasts; thus, the VP3 protein was renamed Apoptin.

Apoptin is a 14kD small molecular protein abundant in basic amino acids like serine and threonine, comprising two nuclear localisation signal sequences (NLS) and one nuclear export signal sequence (NES) (12–14). These domains are vital for Apoptin’s nuclear shuttling. In tumour cells, Apoptin undergoes phosphorylation and subsequently translocates to the nucleus, triggering apoptosis. Conversely, in normal cells, apoptin harbouring NLS may not be adequately activated or could be impeded by inhibitory mechanisms, such as phosphorylation status or chaperone protein binding, preventing an elevation in apoptosis rates. This underscores that mere nuclear localisation alone is insufficient to induce cell death in normal cells (15).

Therefore, in addition to nuclear localisation, the activation of apoptotic proteins also requires one or more additional events, such as the ability of apoptotic proteins to interact with multiple proteins in tumour cells (16), induce tumour cell apoptosis, and exert anti-tumour effects. In this process, they are usually activated by relevant signalling protein pathways in tumour cells, leading to their accumulation in the nucleus, while in normal cells, they remain in the cytoplasm. Elucidating the proteins and signalling pathways involved in the interaction between apoptotic factors and tumour cell apoptosis will contribute to the advancement of targeted tumour therapy mediated by apoptotic factors. Therefore, in addition to nuclear localisation, the activation of apoptotic proteins necessitates one or more supplementary events, such as their capacity to interact with various proteins in tumour cells (16), thereby inducing tumour cell apoptosis and exerting anti-tumour effects. Throughout this process, they are usually activated by pertinent signalling pathways in tumour cells, resulting in their accumulation within the nucleus, whereas in normal cells, they reside in the cytoplasm. Elucidating the proteins and their signalling pathways that interact with apoptins during the apoptosis of tumour cells will facilitate advancements in apoptin-mediated targeted tumour therapy.

Du et al. ^(^ 17 ^)^ efficiently transfected mesenchymal stem cells with apoptin using a lentiviral expression system, enabling them to synthesise and secrete apoptin, which in turn effectively activated caspase-3 and the mitochondrial/cytochrome C signalling pathway, thereby inducing apoptosis in lung cancer cells. Basse et al. (18) demonstrated that, in cervical cancer cell lines, the combination of antimicrobial peptides (ABPs1) and apoptin significantly enhanced caspase-3 activity, suggesting that apoptin induced cell death involves the caspase-dependent mitochondrial pathway. Song et al. (19) validated that recombinant adenovirus carrying apoptotic factors targets AMPK and subsequently inhibits glycolysis, migration, and invasion in lung cancer A549 cells via the AMPK signalling pathway, playing a pivotal role in tumour cell energy metabolism. Zhou et al. (20) demonstrated that apoptotic peptide-derived peptides reverse cancer cell resistance to cisplatin by inhibiting the PI3K/AKT/ARNT signalling pathway, downregulating MDR1 expression, and inhibiting cancer cell invasion and metastasis. In this study, we observed that the recombinant oncolytic adenovirus delivering apoptin induced apoptosis in MCF-7 tumour cells, accompanied by significant alterations in key proteins of the mTOR/S6K signalling pathway. These findings suggest a potential role of this pathway in apoptin-mediated breast cancer cell apoptosis.

Apoptin mainly induces tumour cell death in the form of apoptosis (21), but further research has revealed that apoptin can also promote cancer cell death through other cell death mechanisms or signalling pathways, such as autophagy-dependent death (22) and pyroptosis (23).

Ad-VT (Ad-Apoptin-hTERTp-E1A), a novel oncolytic adenovirus, has the following excellent characteristics. (1) As an oncolytic adenovirus, Ad-VT can replicate in tumour cells and kill tumour cells. (2) The addition of the apoptin gene, namely, the so-called bispecific oncolytic adenovirus, has a targeted killing effect on tumours. (3) In combination with other antitumour drugs or chemotherapy drugs, Ad-VT has a synergistic effect on reducing the toxicity of chemotherapy drugs. (4) Due to its low production- and treatment-related costs, oncolytic adenovirus can be prepared in large quantities, and it is safe and reliable. The purpose of this study was to transfer apoptin to tumour cells to study whether apoptin can promote apoptosis in the human breast cancer cell line MCF-7 *in vivo* and *in vitro*. Apoptin can induce the apoptosis of many types of tumour cells but does not damage normal cells.

A better understanding of the signalling pathway through which apoptin inhibits tumour cell survival is necessary for apoptin to be an effective anticancer drug. In this study, proteomics and Western blot experiments were performed to explore the apoptosis signalling pathway induced by apoptin and the changes in the levels of key proteins.

In this study, MCF-7 tumour cells were labelled with firefly luciferase and inoculated into immunodeficient mice to establish a tumour model. The growth and metastasis of tumour cells in a tumour model can be observed and measured continuously and intuitively using an *in vivo* imaging system for small animals, and even slight changes can be detected, suggesting that these animals constitute an ideal model for more intuitively evaluating the therapeutic effects of anticancer drugs.

## Materials and methods

### Viruses, cells and laboratory animals

Human breast cancer (MCF-7) and Normal human mammary epithelial (MCF-10A) cells were purchased from the Cell Bank of the Shanghai Institute of Biology, whereas human embryonic kidney cells (HEK-293) were purchased from the CCTCC (Cell Bank of the Chinese Academy of Sciences). MCF-7 cells were cultured in RPMI 1640 medium supplemented with 10% foetal bovine serum and 1% penicillin–streptomycin. MCF-10A cells were cultured in DMEM supplemented with 10% foetal bovine serum and 1% penicillin–streptomycin. HEK-293 cells were cultured in DMEM supplemented with 10% foetal bovine serum and 1% penicillin–streptomycin.

Luciferase-labelled human breast cancer cell lines (MCF-7-luc), Ad-hTERTp-E1a-Apoptin (Ad-VT), Ad-Apoptin (Ad-VP3) and Ad-MOCK were constructed and preserved in our laboratory.

In this study, 6–8-week-old female BALB/c nude mice were purchased from Beijing Weitong Lihua Company.

### Methods

#### Amplification and virulence determination of recombinant oncolytic adenovirus

HEK-293 cells were cultured in DMEM supplemented with 10% foetal bovine serum and 1% penicillin–streptomycin. Add 5 × 10^5^ HEK-293 cells to each well of a 6-well cell culture plate and culture them at 37 °C and 5% CO_2_ for subsequent experiments.

(1)Viral amplification

**Figure 1:**
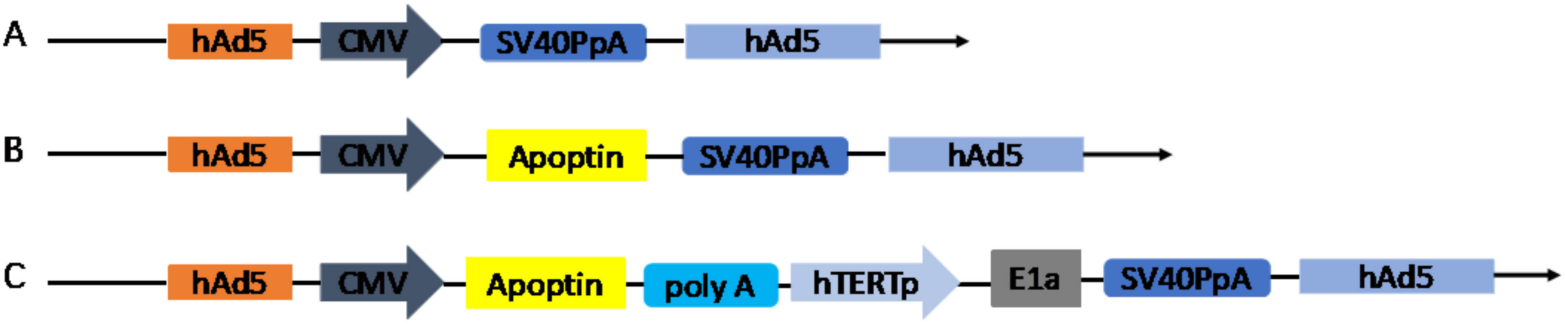
Schematic diagram of four recombinant adenovirus vectors constructed using shuttle vectors in our laboratory: **(A)** MOCK, **(B)** Ad-Apoptin (Ad-VP3), **(C)** Ad-Apoptin-hTERTp-E1a (Ad-VT).

The recombinant adenoviruses (Ad-VT, Ad-VP3 and Ad-Mock) were generated using the RAPAd.I packaging system (**Figure 1**). Ad-VT (Ad-Apoptin-hTERTp-E1A) contains a tumour-specific promoter (hTERTp, human telomerase reverse transcriptase) that activates the E1A gene (essential for viral replication) (24) and a cytomegalovirus (CMV) promoter to initiate the expression of the apoptin gene.

Add 50 moi recombinant oncolytic adenovirus to each well of a 6-well cell culture plate for cultivation until the cell morphology is completely diseased. The cells were collected in culture bottles, repeatedly freeze-thawed to completely break the cells, and stored at -80red

(2)Poison value determination

Perform virus titre determination on the amplified virus, dilute the virus solution in a 1:10 gradient, and transfer it to a 96 well cell culture plate with 5000 HEK-293 cells per well for cultivation. During this period, continuously observe the cell pathology, and observe the pathological phenomenon under an optical microscope for about 6–7 days. The complete pathological morphology of the cells is swelling and rounding, high transparency, and the appearance of floating clusters.

Calculate TCID50 and calculate the PFU value based on the relationship between TCID50 and PFU (Karber formula): lgTCID50=L-d(s-0.5)

L=logarithm of the highest dilution; D=difference between dilution logarithms; S=total positive tube ratio

### MTS assay

MCF-7 and MCF-10A cells were added to 96-well plates (5000 cells/well) for 24 hours. Virus handling: The recombinant adenoviruses Ad-VT, Ad-VP3 and Ad-MOCK were diluted with serum-free medium (MOIs of 50, 100, and 200). The culture medium in the 96-well plate was discarded, diluted recombinant oncolytic adenoviruses (50 μL/well) were added, and the control well was established. The 96-well cell culture plate was removed at 24 h, 48 h and 72 h. The culture medium was discarded, and 110 μL of MTS (Promega, USA)(diluted at 1:10) was added to each well. The mixture was incubated for 80 min in an incubator in the dark. The absorbance of the MCF-7 and MCF-10A cells in each well was measured with an enzyme labelling instrument (Tecan Trading AG, Switzerland) at a wavelength of 490 nm for 20 s, and the cell activity inhibition rates of three recombinant oncolytic adenoviruses and control were calculated. Cell proliferation inhibition rate (%) = (absorbance value of control well-absorbance value of treatment well)/absorbance value of control well × 100%

### Effects of recombinant adenoviruses on the migration of MCF-7 cells

The insert (Culture-Insert 2 Well, ibidi) was placed in a 6-well plate, and then the MCF-7 cell suspension (3.5ens^4^ cells/well) was added. The cells were cultured at 37tC in a 5% CO_2_ incubator until the cells formed a monolayer, after which the insert was removed. The samples were rinsed repeatedly with serum-free RPMI 1640 medium 3 times to ensure that no suspended free cells were present in the scratches, and the initial images were captured with an optical microscope (BX-60, Olympus, Tokyo, Japan). The recombinant adenoviruses Ad-VT, Ad-VP3 and Ad-MOCK were diluted with RPMI 1640 medium, and the PFU was 3.5ium^5^ TCID_50_/100 μ10 After capturing the first photograph, the diluted solution (1 ml, MOI of 5) was added to the 6-well plate and incubated for 2 hours, after which each well was filled with 1 mL of medium. The 6-well plate was removed at 0 h, 8 h, 16 h and 24 h. The culture medium was removed from the 6-well plate and placed in an EP tube before each images was captured and then placed under the microscope as soon as possible. The width of the scratches at different time points was analysed using ImageJ, and the mobility of the MCF-7 cells was calculated as follows: cell mobility = (0 h scratch width-0/8/16/24 h scratch width)/0 h scratch width.

### Observation of the effects of recombinant adenoviruses on the invasion of MCF-7 cells using BioCoat chambers

The MCF-7 cell suspension (2spe^5^ cells/well) was added to a 24-well plate and cultured at 37tC in 5% CO_2_ incubator for 24 hours. For virus treatment, the three recombinant adenoviruses, Ad-VT, Ad-VP3 and Ad-MOCK, were diluted with serum-free RPMI 1640 medium, and the PFU were 2ere^7^ TCID_50_/100 μ10 1100^7^ TCID_50_/100 μ1 and 5nd0^6^ TCID_50_/100 μ10

The culture medium in the 24-well plate was discarded, 250 μ5 (MOIs of 200, 100 or 50) of recombinant adenoviruses was added to the 24-well plate, and a negative control was prepared. After 2 hours of culture in the incubator, 250 μ5 of RPMI 1640 medium was added to each well, and the cells were incubated for 24 hours or 48 hours. The BioCoat (Corning,USA) chambers preserved at -20eC were removed and incubated at room temperature. Serum-free RPMI 1640 medium preheated at 37hC was added to each chamber and hydrated in the incubator for 2 hours. A new 24-well plate was used, 750 μ5 of RPMI 1640 medium was added to each well, sterile tweezers were used to place a BioCoat chamber each well, with one culture plate used for each of the two periods to be tested. The samples from each well were collected in an EP tube by centrifugation at 2000 rpm for 5 min, the liquid was discarded, and 500 μ0 of serum-free RPMI 1640 medium was added to suspend the cells. The samples were transferred to the chamber, and the cells were cultured in an incubator for 24 hours. A new 24-well plate was used, and 825 μ2 of WST-1 (Sigma-Aldrich, USA) (diluted at 1:10) was added to each well. The culture medium in the upper chamber was discarded, the matrix mixture and nonmigrated cells were removed from the small ependyma with cotton swabs, WST-1 was added to the BioCoat chamber, and the 24-well plate was placed into the incubator and incubated for 90 min in the dark. After shaking for 15 s, the OD value of each well was measured with an enzyme labelling instrument at a wavelength of 450 nm.

### Detection of metastasis-related proteins

Suspensions of MCF-7 cells were seeded in a 6-well cell culture plate at a density of 3 × 10^5^ cells/well. The cells were then infected with Ad-VP3 or Ad-VT at an MOI of 100. After 48 hours, we performed Western blotting to determine the levels of key proteins in MCF-7 cells. For this experiment, we incubated membranes containing cell lysates with antibodies against E-cadherin, N-cadherin, SNAIL and vimentin (Cell Signalling Technology, USA)(diluted at 1:1000).

Forty-eight hours after infection, the cells were harvested by centrifugation at 5000 rpm for 5 minutes. Then, 200 μ0 of the cell lysis reagent SD-001 from the MinuteTM Total Protein Extraction Kit (Invent, Germany) was added to the cell pellets. The cell lysates were then homogenised by repeated pipetting and centrifuged at 14000 rpm in a precooled centrifuge tube for 30 sec (4eC). The final concentrations of the protein extracts were then determined using a BCA protein quantification kit (Beyotime Biotechnology, China). The levels of the 4 proteins associated with cell transfer were then analysed using Western blotting.

### Observation of apoptotic bodies via transmission electron microscopy

MCF-7 cells were added to a 6-well plate (2×10^5^ cells/well). After 24 hours of culture, Ad-VT, Ad-VP3 and Ad-MOCK(MOI of 100/well) were added. The control group was established. After 48 hours, the culture medium was removed, and PBS (1×) was added to wash the cells twice. The cells were collected in 15 ml centrifuge tubes. After centrifugation at 1000 rpm for 5 min, 5 mL of PBS (1×) was added to wash the cell precipitate, the mixture was centrifuged at 1000 rpm, and the supernatant was discarded. After 1.5 mL of PBS (1×) was added to the suspended cells for precipitation, the cells were transferred to EP tubes and centrifuged at 1500 rpm for 8 min. Glutaraldehyde (700–800 μL) was slowly added to the precipitate, which was subsequently fixed overnight at 4°C. The samples were washed 3 times with PBS (1×) for 10 minutes each, after which they were fixed again for 2 hours with osmic acid. The samples were washed with PBS (1×) three times for 10 min each, after which they were embedded in epoxy resin overnight. After the samples were cut, the samples were sliced with a 200 mesh copper mesh and stained, and the results were observed via transmission electron microscopy.

### Hoechst staining

MCF-7 cells were added to 6-well plates (2×10^5^ cells/well) and cultured for 24 h, Ad-VT, Ad-VP3 and Ad-MOCK (MOI of 10/well) were added, and control wells were established. One 6-well plate was removed at 24 h, 48 h and 72 h, and the cells in each well were diluted with 1 mL of Hoechst (Life Technologies, USA) dye solution (Hoechst (5 mg/ml) 1:1000 dilution), incubated in an incubator for 15 minutes and washed repeatedly with PBS (1×) for 15 minutes. Then, 500 μL of serum-free medium was added to each well, and the cells were removed, placed on slides, and photographed under the same conditions with a fluorescence microscope (BX-60, Olympus, Tokyo, Japan).

### JC-1 staining

1. Qualitative detection of the mitochondrial membrane potential

After the clean tablets were spread into 6-well plates, MCF-7 cells (2×10^5^ cells/well) were added, and after 24 h of culture, Ad-VT, Ad-VP3 and Ad-MOCK (MOI of 100/well) were added. Control wells were set up, and a 6-well plate was removed at 24 h, 48 h and 72 h after exposure. The medium in the 6-well plate was discarded, 1 mL of JC-1 (Life Technologies, USA) staining solution (diluted 1:1000) was added to each well, and the samples were incubated for 15 min in an incubator in the dark and washed twice with PBS (1×). Five hundred microlitres of serum-free medium was added to each well, and the coverslips were removed and photographed under the same conditions using a fluorescence microscope (BX-60, Olympus, Tokyo, Japan).

2. Quantitative detection of the mitochondrial membrane potential

MCF-7 cells (5,000 cells/well, 100 μL) were added to a 96-well plate and cultured for 24 h, and Ad-VT, Ad-VP3 and Ad-MOCK (MOI of 100, 50 μL) were added to the cells in the virus and control groups. A 96-well plate was removed at 24 h, 48 h and 72 h, and the medium was discarded. Then, 100 μL of JC-1 staining solution (diluted 1:1000) was added to each well, and the samples were incubated for 20 min in an incubator in the dark and washed twice with PBS (1×). After 50 μL of serum-free medium was added, an enzyme labelling instrument (Tecan Trading AG, Switzerland) was used for detection. The detection conditions were as follows: JC-1 monomer, excitation wavelength of 490 nm and emission wavelength of 530 nm; JC-1 polymer, excitation wavelength of 525 nm and emission wavelength of 590 nm.

### Annexin V-FITC/PI detection

1. Laser confocal microscopy observations of fluorescence staining to assess apoptosis

MCF-7 cells (2×10^5^ cells/well) were added to a 6-well plate. After 24 hours of culture, the three recombinant adenoviruses, Ad-VT, Ad-VP3 and Ad-MOCK (MOI of 100), were added, and a control well was established. The cells were collected into EP tubes at 24 h, 48 h and 72 h and centrifuged at 2000 rpm for 5 min. Then, 500 μL of binding buffer (1×) was added to each tube, 5 μL of Annexin V-FITC (Cell Quest Pro, Becton Dickinson) and 5 μL of PI (Cell Quest Pro, Becton Dickinson) were added, and the mixture was incubated for 20 min at room temperature and photographed with a laser confocal microscope(Leica, Germany).

2. Detection of apoptosis by flow cytometry

MCF-7 monollayers were prepared in a 6-well plate with a cell density of 2.0s 105 cells/well and cultured in a 5% CO_2_ incubator at 37c for 24 h. MCF-7 cells were infected with 100 MOI recombinant tumour-lytic adenovirus (Ad-VP3 and Ad-VT).MCF-7 cells were treated with fitc Annexin-V apoptosis kit (Cell Quest Pro, Becton Dickinson) for 24h, 48h and 72h. Meanwhile, negative control group, Annexin V-FITC single staining control group and PI single staining control group were established. Apoptosis was detected by flow cytometry (C6 Plus and FACSCalibur, Becton Dickinson, Franklin Lakes, NJ, USA).

### Proteomics and WB verification of the changes in the levels of key proteins in MCF-7 cells induced by the recombinant oncolytic adenoviruses

1. Proteomic analysis of changes in protein levels

MCF-7 cells were inoculated in 6-well plates (2×10^5^ cells/well). After 24 hours of incubation in the incubator, the cells were inoculated with the recombinant adenoviruses Ad-VT (MOI of 100). 48 hours after recombinant oncolytic adenovirus infection, cells were collected for proteomic analysis, searching for significantly different proteins by using hypergeometric test analysis.

2. Verifying the credibility of the proteomic data via Western blot experiments

The previous experiment was the same as that described in (1). Western blotting was performed to detect the expression levels of the p-mTOR and p-S6K proteins in MCF-7 cells that were incubated with the recombinant adenovirus Ad-VT at an MOI of 100 for 48 hours. Cells that were not infected with the virus during the same period were used as the control group.

### Detection of luciferase activity and stability

MCF-7-luc cells (5×10^3^ cells/well) were added to a 96-well plate and cultured for 48 h. After 3 min, the cells were lysed with an ONE-Glo™ Luciferase Assay System (Promega Corporation, USA)(100 μL/well), and luciferase activity was detected with an enzyme labelling instrument (Tecan Trading AG, Switzerland). The clone with the highest fluorescence value was continuously cultured for 8 weeks, and the luciferase activity was measured using a luciferase detection kit (Promega Corporation, USA) regularly (5 generations) to observe the stable expression of the luc gene.

### *In vivo* imaging of MCF-7-luc cells *in vitro*

The density of the MCF-7-luc cells was diluted in a gradient of 10 dilutions (5×10^5^ cells/mL–9.8×10^2^ cells/mL). A total of 100 μL/well was inoculated into a 96-well plate, and the control group was established. After 24 hours of culture, the fluorescein substrate (Promega Corporation, USA) (luciferin, *in vivo* grade, 150 μg/mL) was added at 100 μL/well, and the relationship between the number of cells and the bioluminescence intensity was analysed.

### Determination of the cell growth curve

MCF-7 cells and MCF-7-luc cells (5000 cells/well) were added to a 96-well plate. One 96-well cell culture plate was removed at 1 d–7 d, and the culture medium was discarded. The growth of the cells was tested with an MTS (Promega Corporation, USA) assay, and the cells were incubated in the dark and shaken for 20 s. The OD values of the two types of cells were measured with an enzyme labelling instrument (Tecan Trading AG, Switzerland) at a wavelength of 450 nm.

### Establishment of a mouse model of breast cancer xenotransplantation

The hormone treatment prior to the formation of breast tumours included an intramuscular (I.M.) injection of oestradiol valerate (2 mg/kg/week) once a week one week before the tumour cell injection. MCF-7-luc cells were collected, the cell density was adjusted to 1×10^7^ cells/mL, and 100 μL of cells was injected into each mouse. After successful tumour cell loading, the mice were randomly divided into 5 groups (Ad-VT treatment group, Ad-VP3 treatment group, Ad-MOCK treatment group, normal saline treatment group and control group), with 6 mice in each group. The size of the tumours was measured every week, and the mice were photographed with a small animal imager (0–6 w). When the tumour has grown to a volume greater than 10mm^3^, treatment is initiated. After continuous treatment for 5 weeks, recombinant adenovirus (1×10^9^ TCID_50_/100 μL) was injected into the tumours every 3 days.

Volume=(Length×Width^2^)/2

### Detection of the luminescence value, tumour volume and life cycle of luciferase in tumour-bearing nude mice

After the tumours were successfully implanted into the nude mice, the tumour site was photographed continuously for 4–6 weeks with an *in vivo* imager (Merck KGaA, Darmstadt, Germany). An intraperitoneal injection of 200 μL of the fluorescein substrate luciferin D-potassium salt (Promega Corporation, USA) (15 mg/mL) was performed in each nude mouse. After 3–5 min, each nude mouse was injected intraperitoneally with 80–100 μL of 1% pentobarbital sodium. After the mice were completely anaesthetised, they were placed in a tray and put into a dark box for imaging (white light photography and bioluminescence photography). The white light image was superimposed on the bioluminescence image, a ruler was added, and the luminescence of the tumour area was calculated to assess the results. The tumour length and width were measured once a week for 5–6 weeks. Survival was recorded every day, and no significant change in the survival of the nude mice in each group (3–5 weeks) was observed.

### Statistical analysis

Statistical significance between groups was determined using GraphPad Prism, Version 8.0 (GraphPad Software, San Diego, CA). Data were presented as mean ± SEM in all experiments and analysed using Student’s t test or one-way analysis of variance (ANOVA) followed by a Dunnett’s *post hoc* test. Comparisons were significant when P < 0.05, P < 0.01 or P < 0.001.

## Results

### Recombinant adenoviruses inhibit the proliferation of MCF-7 and MCF-10A cells

**Figure 2:**
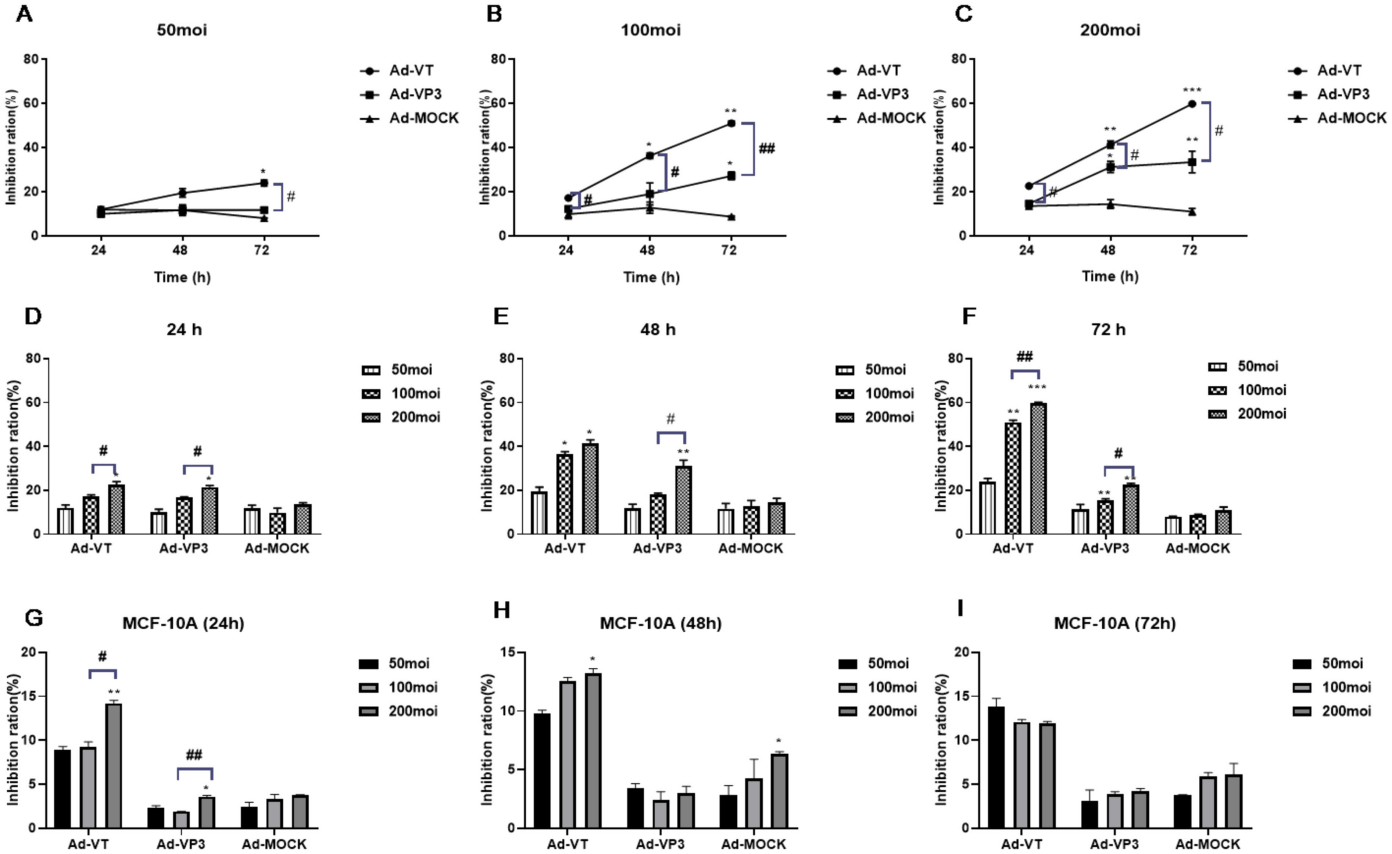
The effect of recombinant oncolytic adenovirus on MCF-7 and MCF-10A cell viability was detected by MTS assay. **(A-C)** Rates at which different concentrations (MOIs of 50, 100, and 200) inhibited MCF-7 cell activity. *P < 0.05 compared with the same recombinant adenovirus group at 24 h, **P < 0.01 compared with the same recombinant adenovirus group at 24 h, and ***P < 0.001 compared with the same recombinant adenovirus group at 24 h. **(D-F)** Inhibitory effects of the recombinant adenoviruses on MCF-7 cell activity at different times (24 h, 48 h, and 72 h). **(G-I)** Inhibitory effects of the recombinant adenoviruses on MCF-10A cell activity at different times (24 h, 48 h, and 72 h). *P < 0.05 compared with the same recombinant adenovirus administered at an MOI of 50, **P < 0.01 compared with the same recombinant adenovirus administered at an MOI of 50, and ***P < 0.001 compared with the same recombinant adenovirus administered at an MOI of 50. (#p<0.05, ##p<0.01, ###p<0.001) when compared with Ad-VT.

The concentration gradient revealed that the inhibitory effects of MOIs of 50, 100 and 200 of Admuri VT on cells significantly increased with increasing infection time, with an obvious effect of time observed in the following order: 72 h > 48 h > 24 h (P<0.05, P<0.01, and P<0.001; **Figures 2A–C**). At 72 h, an MOI of 200 of Ad-VT had the strongest inhibitory effect on MCF-7 tumour cell activity (60.336 ± 0.756%). Because the constructed recombinant Ad-VP3 adenovirus did not have the ability to be continuously amplified, the inhibitory effect on tumour cells was not obvious after 24 hours. When cells were infected with Ad-VP3 for 48 hours, the inhibition of MCF-7 tumour cell activity was the most obvious at an MOI of 200 (26.108 ± 2.005%). Over time, the inhibition rates of Ad-VT and Ad-VP3 on MCF-7 cells increased with increasing concentration (**Figures 2D–F**), with an MOI of 200 > MOI of 100 > MOI of 50. In summary, the inhibitory effects of Ad-VT and Ad-VP3, which carry apoptin, on MCF-7 cells increased with increasing infection time, but the effect of Ad-VP3 began to decrease at 72 h. A dose–effect relationship was observed between Ad-VT and Ad-VP3 (MOI of 200>MOI of 100>MOI of 50), and the strength of the inhibitory effect was as follows: Ad-VT>Ad-VP3. For normal mammary epithelial cells MCF-10A, three recombinant adenoviruses at three time periods of 24, 48h, 72h and three different concentrations of 50MOI, 100MOI and 200MOI did not show significant cell inhibition, and the inhibition rate was less than 15% (**Figures 2G–I**).

### Recombinant oncolytic adenoviruses inhibit the migration and invasion of MCF-7 cells

**Figure 3:**
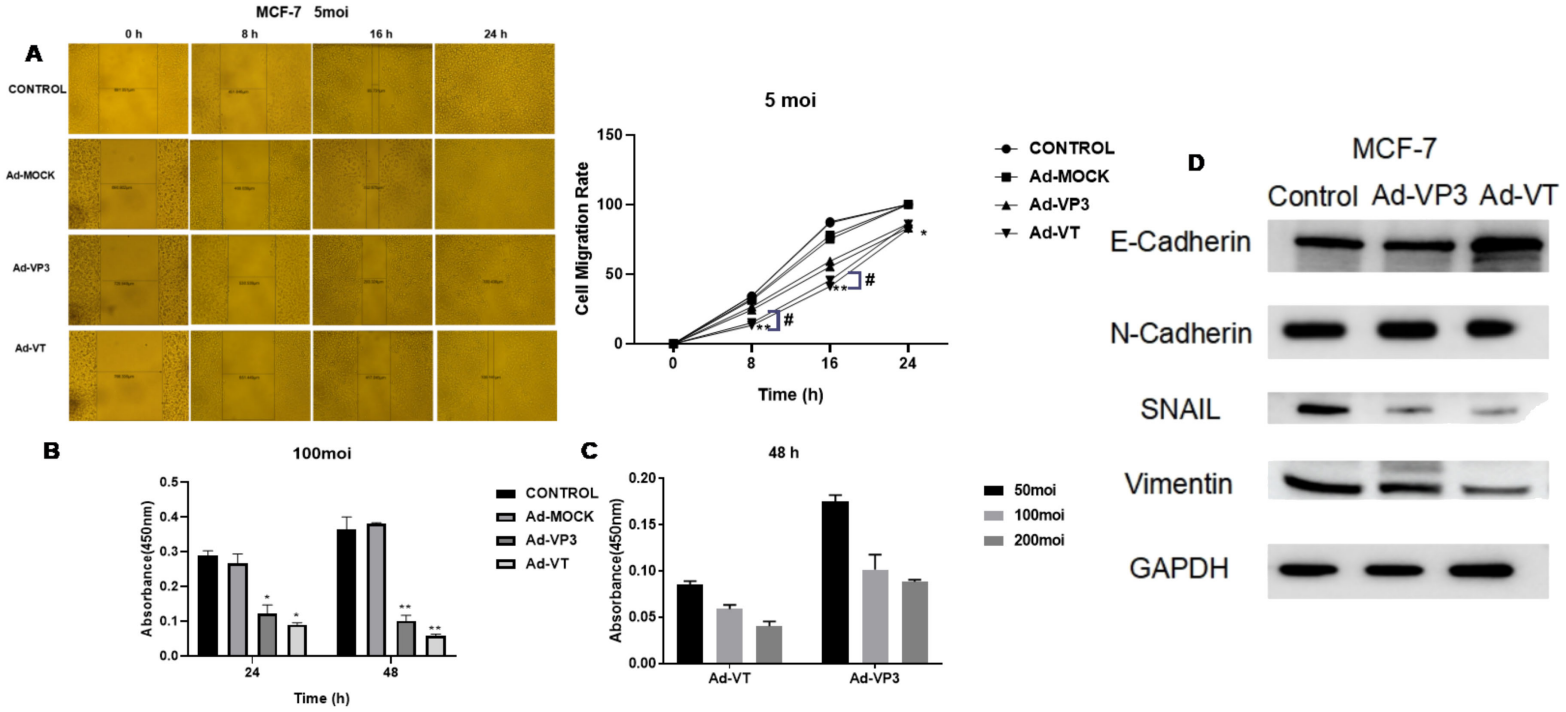
Effects of the recombinant oncolytic adenoviruses on the migration and invasion of MCF-7 cells detected using migration and BioCoat™ Matrigel^®^ invasion chamber assays. **(A)** The administration of three recombinant oncolytic adenoviruses at an MOI of 5 (Ad-VT, Ad-VP3, and Ad-MOCK) inhibited MCF-7 cell migration. **(B)** Three recombinant oncolytic adenoviruses were used to infect MCF-7 cells at an MOI of 100, and invasion was detected after 24 and 48 hours. All the measurements were performed in triplicate, and the means ± standard deviations were compared with those of the control group (*P<0.05 and **P<0.01). **(C)** After infecting MCF-7 cells with different concentrations of recombinant adenoviruses (MOIs of 50, 100, or 200) for 48 h, the invasion results were detected with a BioCoat™ Matrigel^®^ invasion chamber. **(D)** After 48 hours, we extracted total protein from the cells and performed Western blotting to detect the protein expression levels of E-cadherin, N-cadherin, SNAIL and vimentin. All measurements were performed in triplicate, and the means ± standard deviations were compared with those of the MOI group (*P<0.05 and **P<0.01). MOI, multiplicity of infection. #p<0.05 when compared with Ad-VT.

Images were captured at the same location at 0 h, 8 h, 16 h and 24 h after scratch formation (**Figure 3A**). The migration rate of cells infected with Ad-VT and Ad-VP3 was significantly lower than that of the Ad-Mock group and Control group, and Ad-VT had the strongest inhibitory effect on cell migration. At 8 h, the migration rate of the MCF-7 cells was 15.01%. The migration rate of MCF-7 cells in the Ad-MOCK VP3 group was 26.5%, which was significantly lower than that in the Ad-MOCK group (32.27%) and the control group (34.26%). The inhibitory effects of the three recombinant adenoviruses on MCF-7 cell migration were in the following order: Ad-VT > Ad-Vp3 > Ad-Mock.

Both Ad-VT and Ad-VP3 inhibited the invasion of MCF-7 cells (**Figure 3B**). Compared with that of the control, the inhibitory effect of Ad-VT on invasion was greater than that of Ad-VP3 at 24 h or 48 h (P < 0.001) and was time-dependent because the value measured at 48 h was greater than that at 24 h. However, Ad-MOCK had no inhibitory effect on the invasion of MCF-7 tumour cells. In addition, at 48 h, the inhibitory effects of Ad-VT and Ad-VP3 on cell invasion were as follows: MOI of 50 < MOI of 100 < MOI of 200 (**Figure 3C**). The invasion rates of the three recombinant adenoviruses were in the following order: Ad-VT < Ad-VP3 < Ad-Mock. Ad-VT and Ad-VP3, which carry apoptin, also exhibited a time-dependent relationship. The invasion rate of cells infected for 48 hours was lower than that of cells infected for 24 hours.

Western blotting was performed to detect changes in the ability of the recombinant oncolytic adenoviruses to induce MCF-7 cell-associated metastasis (**Figure 3D**). After 48 hours of stimulation, the recombinant oncolytic adenoviruses increased the expression of the E-cadherin protein and decreased the expression of the HIF-1, VEGF-C, MMP-3, MMP-9, N-cadherin, SNAIL and vimentin proteins in MCF-7 cells. The above results showed that recombinant oncolytic adenoviruses could inhibit the migration and invasion of breast cancer cells, reduce the expression of metastasis-related proteins, and reduce the metastatic ability of breast cancer cells.

### Observation of apoptotic bodies via transmission electron microscopy

**Figure 4:**
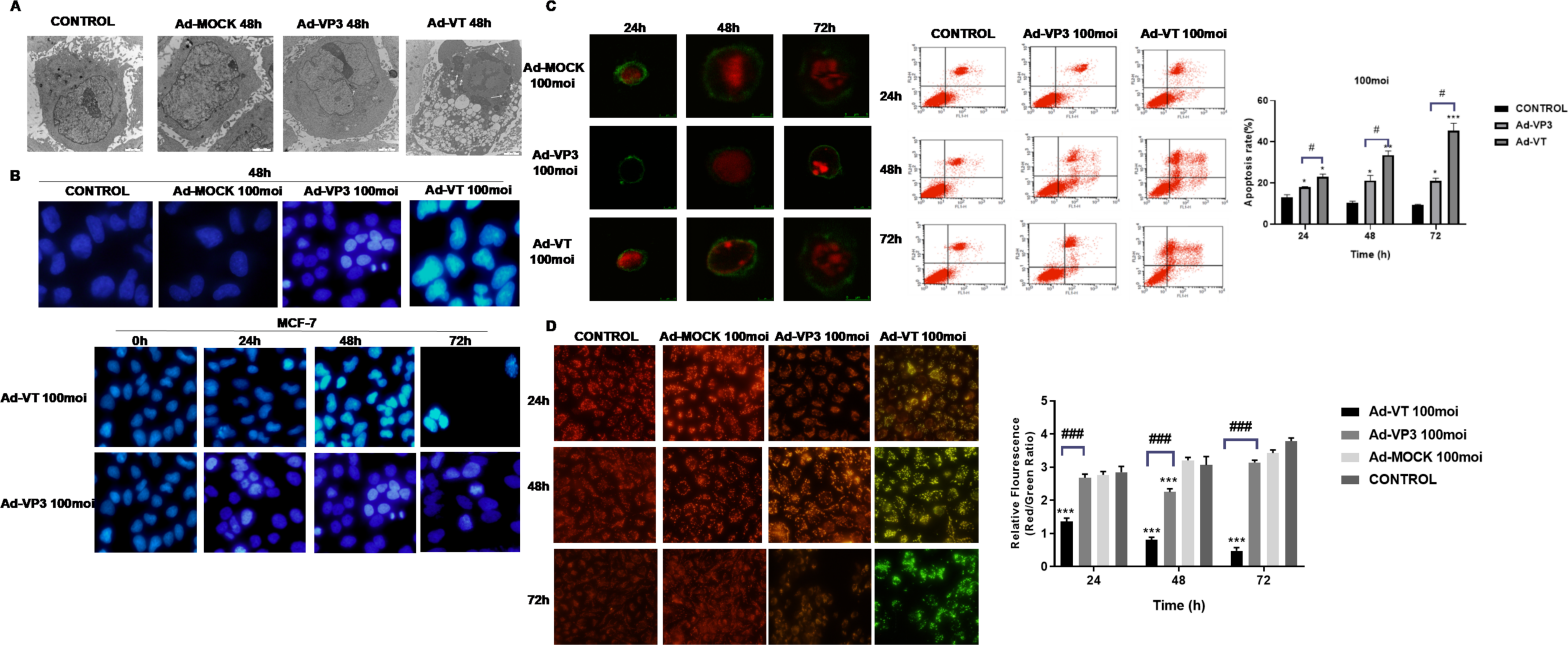
The apoptosis of MCF-7 cells induced by recombinant adenoviruses was detected via transmission electron microscopy, Hoechst staining, JC-1 staining, Annexin V staining, laser confocal microscopy and flow cytometry. **(A)** TEM revealed apoptotic structures in the enlarged images of cells treated with Ad-VT, Ad-VP3 or Ad-MOCK for 48 h. **(B)** MCF-7 cells were treated with recombinant oncolytic adenoviruses, and changes in the morphology of breast cancer cell nuclei were observed via Hoechst staining at 24 h, 48 h, and 72 h. **(C)** Apoptotic breast cancer cells were analysed by laser confocal microscopy and flow cytometry after staining with Annexin-V FITC/PI at 24 h, 48 h and 72 h. **(D)** Changes in the mitochondrial membrane potential of breast cancer cells induced by recombinant oncolytic adenovirus were analysed via JC-1 staining. All the measurements were performed in triplicate, and the means ± standard deviations were compared with those of the control group (*P<0.05 and ***P<0.001). (#p<0.05 and ###p<0.001) when compared with Ad-VT.

Apoptotic bodies were distributed in MCF-7 cells treated with Ad-VT (MOI of 100) (**Figure 4A**). Typical apoptotic bodies were also detected in Ad-VP3-infected MCF-7 cells. Compared with those in the control group and Ad-MOCK group, nuclear fragments and apoptotic bodies formed by nuclear fragmentation were observed 48 hours after Ad-VT and Ad-VP3 infection, especially in the Ad-VT group.

### Changes of nucleus induced by recombinant oncolytic adenovirus in breast cancer

After the MCF-7 cells were infected with Ad-VT and Ad-VP3 (**Figure 4B**), the nuclear staining was uneven, some of the nuclei presented bright blue staining, and some cell swelling or nuclear fragmentation was observed in the Ad-VT group, whereas the nuclei of the Ad-MOCK group and control group presented uniform blue fluorescence, indicating that apoptosis occurred in both the Ad-VT and Ad-VP3-infected cells and that the degree of apoptosis was Ad-VT > Ad-VP3. In addition, we observed cells at different time points (0 h, 24 h, 48 h, and 72 h) and found that the nuclei of MCF-7 cells infected with Ad-VT became increasingly brighter with prolonged treatment (72 h > 48 h > 24 h > 0 h) in a time-dependent manner (**Figure 4B**).

### Recombinant oncolytic adenovirus induces apoptosis of breast cancer cells

After the MCF-7 cells were infected with the recombinant adenoviruses Ad-VT and Ad-VP3 for 48 h and 72 h, the cells with green fluorescence on the cell membrane and red fluorescence in the nucleus were detected via laser confocal microscopy, and some of them exhibited typical characteristics of apoptosis (**Figure 4C**): cell membrane blebbing, cell membrane damage, nuclear fragmentation, and cell swelling. No obvious characteristics of apoptotic cells were observed in the Ad-VP3 group at 24 h. Confocal laser scanning microscopy revealed that Ad-VT- and Ad-VP3-infected MCF-7 cells underwent apoptosis in a time-dependent manner: 72 h > 48 h > 24 h. However, a very small number of apoptotic cells was occasionally observed in the control group and Ad-MOCK group, which were difficult to capture via laser confocal microscopy. We further observed the apoptosis of MCF-7 cells induced by apoptin and found that Ad-VT and Ad-VP3, which carry apoptin, could induce the apoptosis of MCF-7 cells at three different time points (24 h, 48 h and 72 h) (**Figure 4C**). However, the degree of apoptosis was different at different time points as follows: Ad-VT > Ad-VP3, 72 h > 48 h > 24 h. The apoptosis rate in the Ad-VT and Ad-VP3 groups was significantly higher than that in the control group at 48 h and 72 h, and the apoptosis rate reached a maximum at 72 h as the duration of Ad-VT treatment increased. The rate of apoptosis induced by Ad-VP3 was also significantly higher than that in the control group [48 h group (23.268 ± 1.103)% vs. (10.680 ± 1.104)%, P < 0.05; 72 h group (22.002 ± 0.317)% vs. (9.670 ± 1.102), P < 0.05; **Figure 3C**]. The rate of apoptosis induced by Ad-VP3 was the highest at 48 h and decreased at 72 h. We further observed concentration dependence of the effect and found that the concentration was positively correlated with the degree of apoptosis, and the apoptosis rate of MCF-7 cells treated with Ad-VT for 48 h changed in the order of MOIs of 200 > 100 > 50. These results suggest that Ad-VT and Ad-VP3 induce apoptosis by causing changes in the cell membrane.

### Recombinant oncolytic adenovirus induces apoptosis by changing mitochondrial membrane potential of breast cancer cells

MCF-7 cells were inoculated with the recombinant adenoviruses Ad-VT, Ad-VP3 and Ad-MOCK. After 24, 48 and 72 hours of treatment, the inhibitory effects of Ad-VT and Ad-VP3 on MCF-7 cells were determined by monitoring changes in the mitochondrial membrane potential (**Figure 4D**). The three recombinant adenoviruses had different abilities to induce apoptosis and different levels of membrane depolarisation. The ability of Ad-VT to induce apoptosis increased with increasing infection time, and the number of apoptotic cells increased gradually. JC-1 gradually changed from an initial red aggregate to a green monomer. Compared with the control group, the Ad-VP3 group also presented obvious colour changes, but the colour comparison was not obvious at different time points. The ability of Ad-VT to induce apoptosis in MCF-7 cells was greater than that of Ad-VP3 (**Figure 4D**), the number of apoptotic cells was greater at 72 h in the Ad-VT group, and the ratio of red fluorescence to green fluorescence was significantly lower than that in the control group (P < 0.001). The ability of Ad-VP3 to induce apoptosis was greater than that of the control group (P < 0.01), as the ratio of red fluorescence to green fluorescence decreased; however, because Ad-VP3 could not replicate and proliferate, the ratio of red fluorescence to green fluorescence increased at 72 h. At any of the three different time points, the ratio of red fluorescence to green fluorescence in the infected cells was in the order of Ad-VT < Ad-VP3 < Ad-MOCK.

### Changes of apoptosis-related protein expression levels induced by recombinant oncolytic adenovirus in breast cancer cells

**Figure 5:**
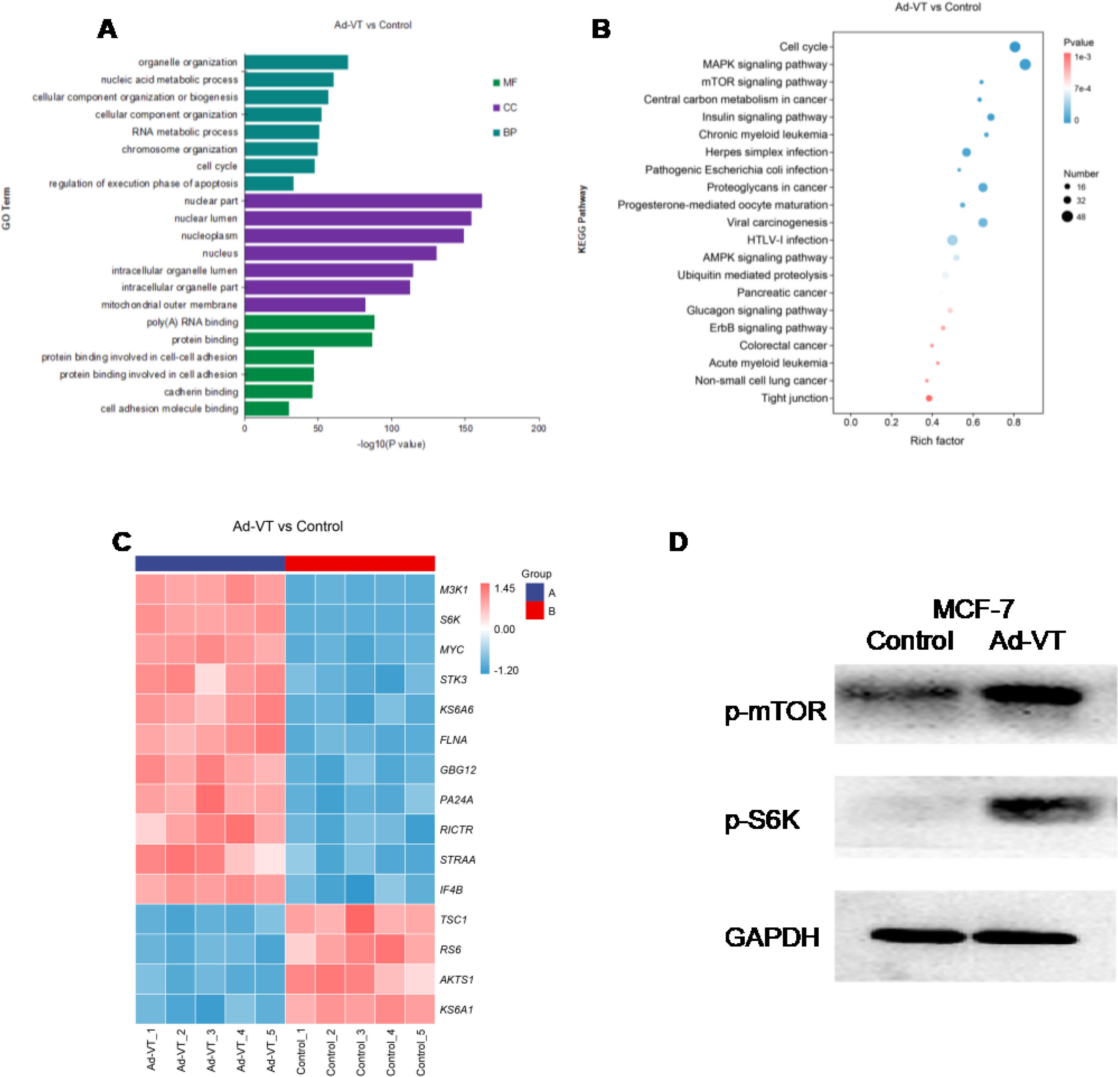
Proteomic analysis of the effects of recombinant adenoviruses carrying apoptin on MCF-7 cells. **(A)** MCF-7 cells were infected with recombinant oncolytic adenoviruses (Ad-VT ) for 48 h, and the changes in cell biological processes, cell components and molecular functions were analysed via proteomics. **(B)** After infecting MCF-7 cells with recombinant oncolytic adenoviruses (Ad-VT ) for 48 h, the differentially expressed genes were subjected to KEGG analyses. **(C)** After recombinant oncolytic adenovirus (Ad-VT) infected MCF-7 cells for 48 hours, Ad-VT induced MCF-7 cells to express differential genes. **(D)** After infecting MCF-7 cells with recombinant oncolytic adenoviruses for 48 h, the changes in the levels of three proteins were analysed by Western blotting. All measurements were performed in triplicate.

Proteomic analysis revealed that, compared with control cells, MCF-7 cells infected with recombinant adenovirus (Ad-VT) presented significant differences in multiple signalling pathways and protein expression. Forty-eight hours after exposure, GO enrichment analysis revealed that the q values of the genes associated with organelles organization and nucleic acid metabolic processes were small (close to 0), and the difference was significant. Among the cell components, the nuclear part and nuclear lumen genes had significant changes, and the differences were significant. In molecular function, the q values of poly(A) RNA binding and protein binding genes were small and close to 0, and the differences of genes were significant(**Figure 5A**). According to the functional annotation and classification results of differential genes, we conducted enrichment analysis of the KEGG pathway(**Figure 5B**), and found that the Rich factor value of the signalling pathway with changes in MAPK and mTOR pathways gradually increased, and the closer Pvalue was to 0, it showed that the enrichment degree gradually increases and the enrichment becomes more obvious. Subsequently, differential protein heat maps showed that M3K1, S6K, MYC, STK3, KS6A6, FLNA, GBG12, PA24A, RICTR, STRAA and IF4B were highly expressed in MCF-7 cells after infection with Ad-VT, while TSC1, RS6, AKTS1 and KS6A1 were low(**Figure 5C**). According to the results of KEGG analysis, cell cycle, MAPK, mTOR pathway and other top ranked pathways are related to apoptosis, and S6K was found to have a significant difference in differential proteins, and S6K is a key gene in the mTOR pathway. Some studies have shown that activation on the mTOR pathway can cause apoptosis, in which S6k activation plays an important role (7, 8). Therefore, we suspect that the elevation of S6K is the key to promoting apoptosis.

In order to determine the proteomic results(**Figure 5D**), WB detection was performed after infection with Ad-VT, and it was found that phosphorylated mTOR was significantly up-regulated at 48h after Ad VT was treated on MCF-7 cells compared with the control group, phosphorylated S6K proteins was upregulated at 48h. The above results are consistent with the results of labelfree proteomic experiments, namely, the promotion of apoptosis of MCF-7 cells by recombinant adenovirus Ad-VT carrying apoptin may be related to the increase of S6K. In summary, the results of protein immunoblotting are basically consistent with the quantitative results of proteomic experiments, with good repeatability.

### MCF-7-luc cells can stably express luciferase

**Figure 6:**
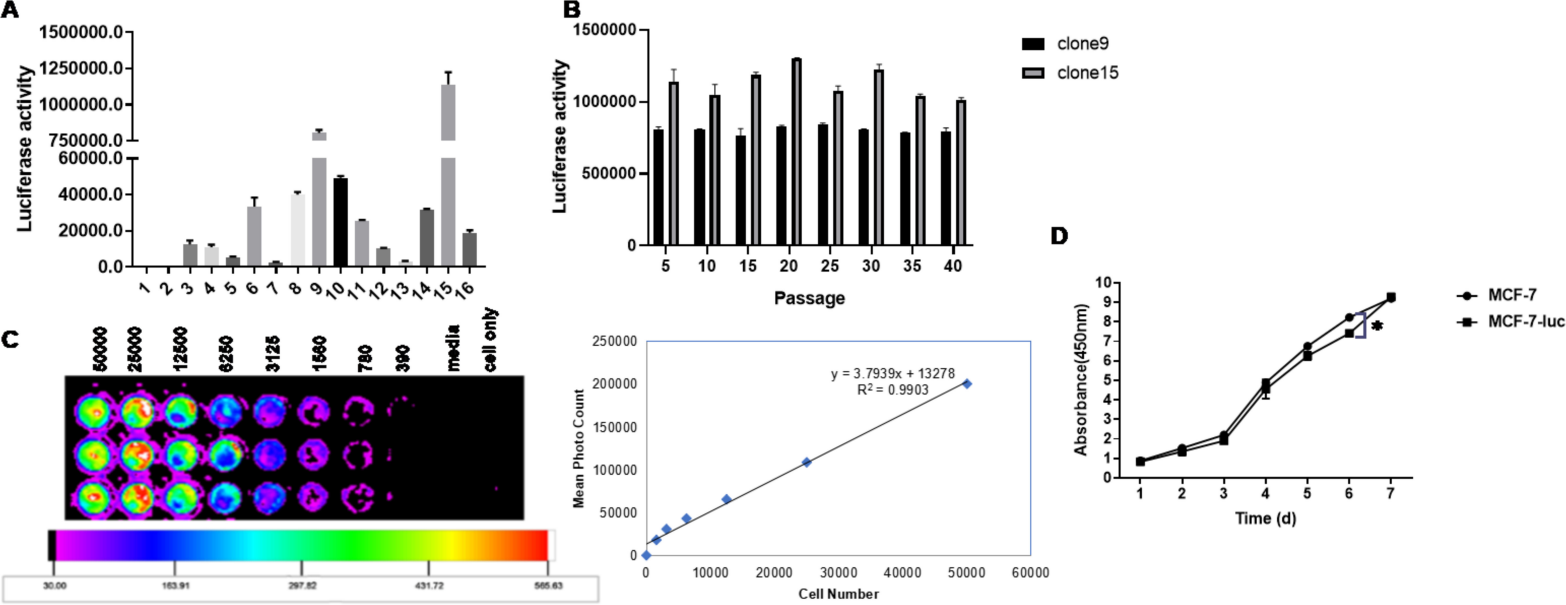
Enzyme activity of luciferase in a human breast cancer cell line (MCF-7-Luc) and comparison of their biological characteristics before and after the test. **(A)** Luciferase activity of different cell clones. **(B)** Luciferase stability in MCF-7-luc cells from different generations. **(C)** MCF-7-luc cells were inoculated into 96-well plates according to the principle of multiple dilutions. After the addition of luciferase, the luminescence intensity was observed. The luminescence intensity increased as the number of cells increased. **(D)** MCF-7 and MCF-7-luc cells were cultured in a 96-well plate. The cell growth rates were detected on days 1, 2, 3, 4, 5, 6 and 7. All the measurements were performed in triplicate, and the means ± standard deviations were compared with those of the MCF-7-luc group (*P<0.05).

A luciferase assay system was used to detect the luciferase activity of different cell clones (**Figure 6A**). Among the 15 groups of cloned cell lines, the luciferase activity of Clones 9 and 15 was the highest.

The two selected clones with the highest fluorescence values were amplified and cultured to observe whether passage had any effect on luciferase activity. The luciferase activity was measured every 5 generations, and no significant change in the fluorescence value at the initial detection up to the 40th generation was detected (**Figure 6B, P** > 0.05), which showed that the two screened cell lines did not affect the luciferase activity due to cell passage and still maintained a stable and high level of luciferase activity.

Clone 15 was seeded in 96-well plates at a density of 50,000 cells/100 μl in the first row of wells. The cell concentration was gradually reduced by double dilution, and then, fluorescein substrate was added and detected with a small animal living imager. The bioluminescence intensity of the MCF-7-luc cells was proportional to the number of cells, with a correlation coefficient of R^2^ = 0.9903, and no fewer than 400 cells were detected (**Figure 6C**). MCF-7-luc cells expressing the firefly luciferase gene were successfully generated from this cell line.

### Comparison of the growth curves of MCF-7 cells and McF-7-luc cells

The growth trends of MCF-7 cells and McF-7-luc cells were not significantly different, and the growth curves were basically consistent (**Figure 6D**), indicating that the growth characteristics of McF-7-luc cells expressing the firefly luciferase gene were not significantly different from those of human MCF-7 breast cancer cells.

### Recombinant oncolytic adenovirus inhibited tumour bioluminescence intensity

**Figure 7:**
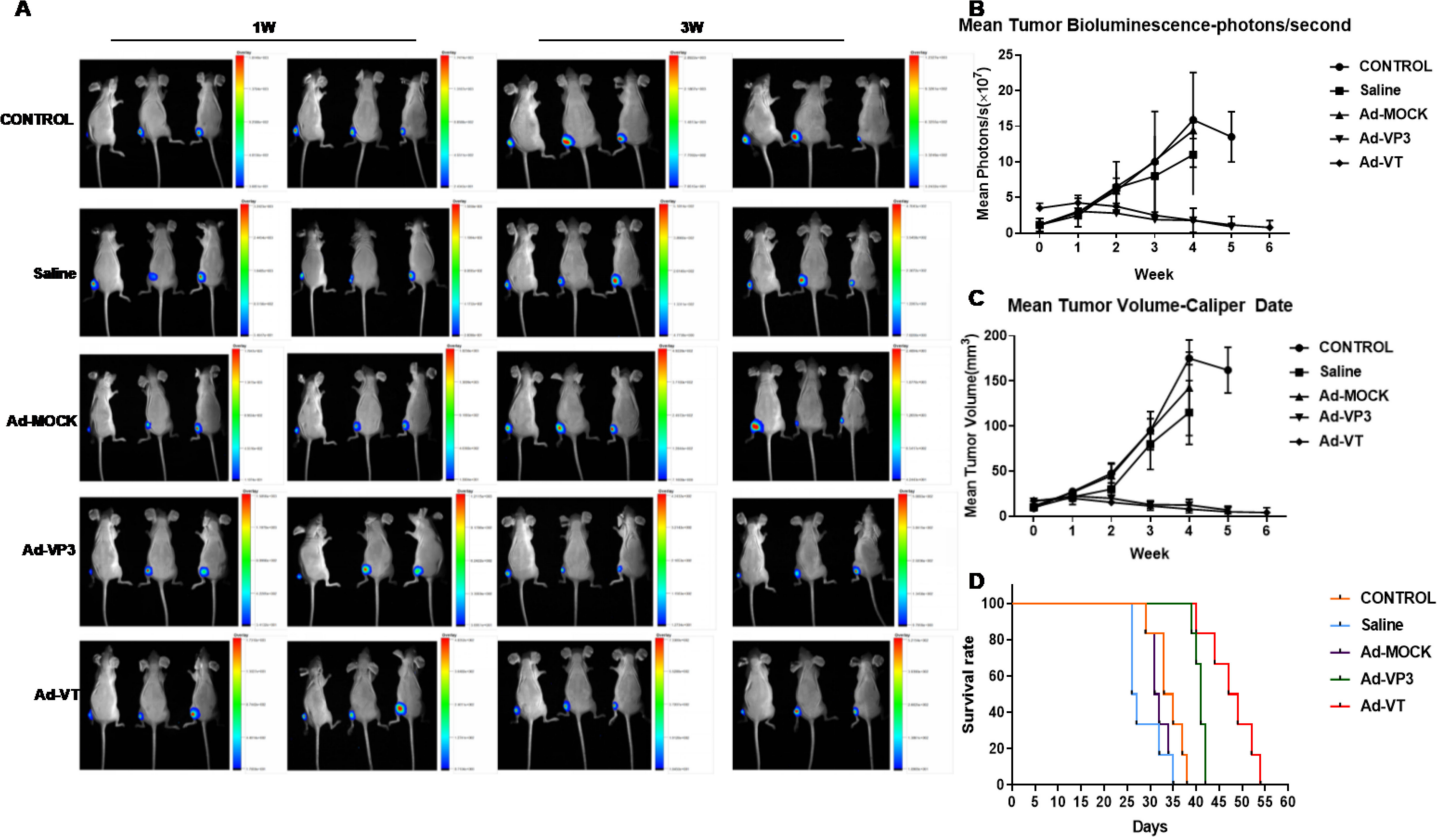
Effect of the recombinant oncolytic adenoviruses on breast cancer in a BALB/c nude mouse model. **(A, B)** MCF-7-luc cells (1×10^7^/100 litres) were injected subcutaneously into the near back of the right hind limb of the mice (6 mice in each group) to establish the xenograft model. An in vivo imaging luminescence system was used to monitor changes in tumour bioluminescence intensity continuously. **(C)** After the xenograft model was successfully established in nude mice, the survival of the mice was recorded daily for 5 weeks. Vernier callipers were used to measure the diameter of the tumours in the nude mice, the tumour volume was calculated once a week, and continuous measurements were performed for five weeks. **(C)** The average tumour inhibition rate of the 1×109 PFU/100 µL Ad-VT treatment group was significantly higher than that of the other groups. The survival rate of the 1×10^9^ PFU/100 µL Ad-VT treatment group was also the highest, and the average survival rate of the nude mice exceeded 60%. **(D)**The survival time of the 1×10^9^ PFU/100 µL Ad-VT treatment group was significantly prolonged compared with the control group or the 1×10^9^ PFU/100 µL MOCK treatment group.

Female BALB/c (nu/nu) mice were subcutaneously inoculated with MCF-7-luc cells, and the bioluminescence intensity of the tumour cells was observed with a small animal *in vivo* imager beginning at 0 W after inoculation (**Figures 7A, B**). We found that the average bioluminescence intensity of the control group, the normal saline treatment group and the Ad-MOCK treatment group increased weekly, and the intensity increased rapidly. The luminescence intensity of the three groups was essentially the same at 0–2 w, and the average luminescence intensity of the normal saline treatment group began to be slightly lower than that of the control group and the Ad-MOCK treatment group beginning in the 3rd week; however, no significant difference in the average weekly bioluminescence intensity was observed among the three groups (P > 0.05). The average bioluminescence intensity of the Ad-VT and Ad-VP3 treatment groups decreased slowly, and the average bioluminescence intensity of the two groups was not significantly different (P > 0.05). Starting from the third week of photography, namely, the first week after treatment, the luminescence intensity of the Ad-VT treatment group and the Ad-VP3 treatment group differed from that of the control group and gradually increased (**Figure 6B**), and significant differences were observed at 3 w and thereafter (P < 0.05).

### Recombinant oncolytic adenovirus inhibited tumour growth in BALB/c nude mice

After the MCF-7-luc cells were inoculated subcutaneously near the backs of the hind limbs of the nude mice, the tumour size was measured once a week for 4–6 weeks (**Figure 7C**). The tumour volumes of the control group, the normal saline group and the Ad-MOCK treatment group all increased significantly over time, increasing from approximately 10 mm^3^ to more than 100 mm^3^ at 4 w, and no significant difference in the average tumour growth rate was observed among the three groups (P > 0.05, **Figure 7C**). Neither normal saline nor Ad-MOCK inhibited tumour growth, but the tumour volume of the Ad-VT treatment group and the Ad-VP3 treatment group tended to decrease after treatment (**Figure 7C**), and the rate of reduction in the average tumour volume of the two treatment groups was slower than that of the control group. We found that before treatment (0 W and 1 W), the average tumour volume of the five groups of mice was not significantly different, but after different toxic treatments (2 W), the tumour volume began to change differently. Due to the different trends, the tumour volume of the two treatment groups, Ad-VT and Ad-VP3, decreased, whereas the tumour volume of the other three groups increased continuously, and the differences between them became increasingly obvious. In summary, Ad-VT and Ad-VP3, which carry apoptin, have inhibitory effects on the MCF-7 human breast cancer cell line.

### Survival curves of tumour-bearing BALB/c nude mice after treatment

After the subcutaneous inoculation of MCF-7-luc cells into BALB/c female mice, the survival times of the nude mice in the different groups were observed (**Figure 7D**). The mice in the saline group died at approximately 25 days, and the mice in the control group and the Ad-MOCK treatment group died at 29 days. In the next 10 days, 6 mice in each of the three groups died, and the average survival time was 34.17 days in the control group, 28.67 days in the normal saline group, and 32.00 days in the Ad-MOCK treatment group. No significant difference in mean survival was observed between the Ad-MOCK group and the control group (P > 0.05). The first mouse in the Ad-VP3 group and Ad-VT group died at approximately the 40th day, and the average survival times were 40.83 d and 47.67 d, respectively. The survival time of both groups was significantly longer than that of the control group; in particular, the survival time of the Ad-VT group was longer than that of the Ad-VP3 group. The survival rate of both groups was 100% after 38 days of tumour cell inoculation, and all of the mice in the control group died. These results indicated that the Ad-VP3 and Ad-VT treatments significantly prolonged the average survival time and improved the survival rate of tumour-bearing mice.

## Discussion

Breast cancer is the most common type of cancer among women, and its incidence is increasing worldwide. Each year, approximately 1400000 women are diagnosed with breast cancer, and approximately 500000 people die (25). Breast cancer has become the tumour with the highest incidence worldwide (26–28). At present, surgical treatment for primary breast cancer is combined with targeted therapy or chemotherapy, endocrine therapy, and immune therapy (3, 29). With continuous improvements in technology, these treatments have achieved good curative effects, but the current treatment strategies for patients still have greater side effects, with a certain negative effect on patients’ quality of life.

Gene therapy is one of the most promising cancer treatments and is now in a stage of rapid development. The selection of appropriate therapeutic genes is the first stage in the development of gene therapy. Due to its potential application for treatment with a variety of genes, adenovirus is a promising tool for cancer treatment because of its ability to manipulate genes and exert a variety of anticancer effects (30–33). Apoptin, a highly conserved protein derived from chicken anaemia virus (CAV), can selectively induce the apoptosis of many malignant tumour cells without affecting normal cells and has become an important latent gene therapy for cancer (34–37). Apoptin is the first isolated tumour-selective anticancer gene. The independence of the small virus protein p53 in tumour cells can be induced by specific types of cell death (21, 38–40). Our laboratory developed a method of gene therapy to observe the effects on apoptosis and the mechanism of action in MCF-7 human breast cancer cells and to inhibit tumour growth *in vivo* and *in vitro*.

Oncolytic viruses (OVs), which evolved and are engineered for cancer specificity, are gaining momentum as a new class of drugs in the fight against cancer. This research used the MTS test to preliminarily evaluate the effects of recombinant adenovirus infection on the proliferation of MCF-7 cells. Both Ad-VT and Ad-VP3 had significant inhibitory effects on MCF-7 cells because Ad-VT not only carried apoptin but also carried a peculiar tumour promoter (hTERTp, human telomerase reverse transcriptase) and expressed the E1A gene (viral replication essential gene). After infection in breast cancer cells, replication and amplification, the strength of the MTS signal within 24 h is significantly greater than that of Ad-VP3 infection after 72 h. Ad-VT (200 MOI) inhibited the proliferation of MCF-7 cells with a strength of 60.34% and was positively correlated with the time and dose. Ad-VP3 was not as potent as Ad-VT in MCF-7 cells because of its lack of a sustained expansion capacity and its effect was not time sensitive; however, it also affected MCF-7 cells with increasing infectious concentrations, and the inhibition rate was significantly increased. Ad-MOCK had no significant inhibitory effect on MCF-7 cells.

After MCF-10A cells were infected with Ad-VT, the inhibition rate was less than 15%. Although the inhibition rate was very low, it could not completely damage normal mammary epithelial cells. Although Ad-VT has been genetically modified to become a bispecific oncolytic adenovirus, its targeting is still insufficient, which also reflects the limitations of oncolytic adenovirus as a viral therapy.

Ya Cao et al. reported that CYT997 induces autophagy and apoptosis in gastric cancer by triggering mitochondrial ROS accumulation to silence the JAK2/STAT3 pathway. In another study, Wenjin Liang et al. suggested that miR-644a promotes apoptosis in HCC cells by inhibiting HSF1. This study aimed to further clarify the effects of this virus on promoting the apoptosis of MCF-7 cells 48 hours after Ad-VT (MOI of 100) or Ad-VP3 (MOI of 100) infection, and transmission electron microscopy was used to observe typical cytoplasmic organelles and nuclear fragments of the apoptotic body, confirming that the recombinant adenoviruses can cause MCF-7 cell apoptosis. By performing Annexin V-FITC/PI staining, flow cytometry and laser confocal microscopy experiments, the three recombinant adenoviruses, Ad-VT, Ad-VP3 and Ad-MOCK, were found to be effective in the treatment of MCF-7 cells at 48 h after infection. Both Ad-VT and Ad-VP3 induced obvious apoptosis in MCF-7 cells, with Ad-VT > Ad-VP3 and an MOI of 200>an MOI of 100>an MOI of 50. However, the cell apoptosis rate was not consistent with the results of the cell inhibition rate. The apoptosis rate induced by Ad-VT at an MOI of 100 (48 h) was 37% higher than the cell suppression rate of 33%. At 72 hours, Ad-VT (MOI of 100) produced a 45% lower apoptosis rate than the cell inhibition rate of 51%, and the difference between the two may be due in part to the use of different assays. Additionally, other death modalities are likely involved.

The results of the JC-1 assay revealed that the ability of Ad-VT to induce apoptosis gradually increased with increasing infection time, as JC-1 gradually changed from an initial red aggregate to a green monomer. Ad-VP3 also obviously changed the colour of JC-1 compared with that of the control group; moreover, no significant difference was observed between the Ad-MOCK group and the control group. Compared with the results of the control group, the results of standard fluorescence microscopy were the same: in the Ad-VT group (the ratio of red fluorescence to green fluorescence), the ratio was the lowest (P < 0.001). Compared with the control group, Ad-VP3 induced apoptosis in MCF-7 cells at 48 h.

Hui Zeng et al. showed that lycorine induced apoptosis of A549 cells by regulating AMPK-mTOR-S6K signalling pathway (8). The proteomic analysis revealed that the phosphorylated mTOR protein may be involved in apoptin-induced apoptosis in MCF-7 cells, especially changes in the p-S6K proteins associated with the p-mTOR protein, which provides a new approach for studying the potential signalling pathways involved in apoptin-induced apoptosis.

This study conclusively demonstrates that oncolytic adenovirus-delivered apoptin effectively induces apoptosis in MCF-7 breast cancer cells, with the apoptin response exhibiting dose- and time-dependent characteristics. Label-free quantitative proteomic analysis revealed significant alterations in both the global proteome and phosphoproteome of Ad-VT-infected MCF-7 cells. KEGG pathway enrichment analysis of differentially expressed proteins identified the MAPK and mTOR signalling pathways as being particularly prominent, showing substantial enrichment and marked changes in associated pathway proteins.

**Figure 8:**
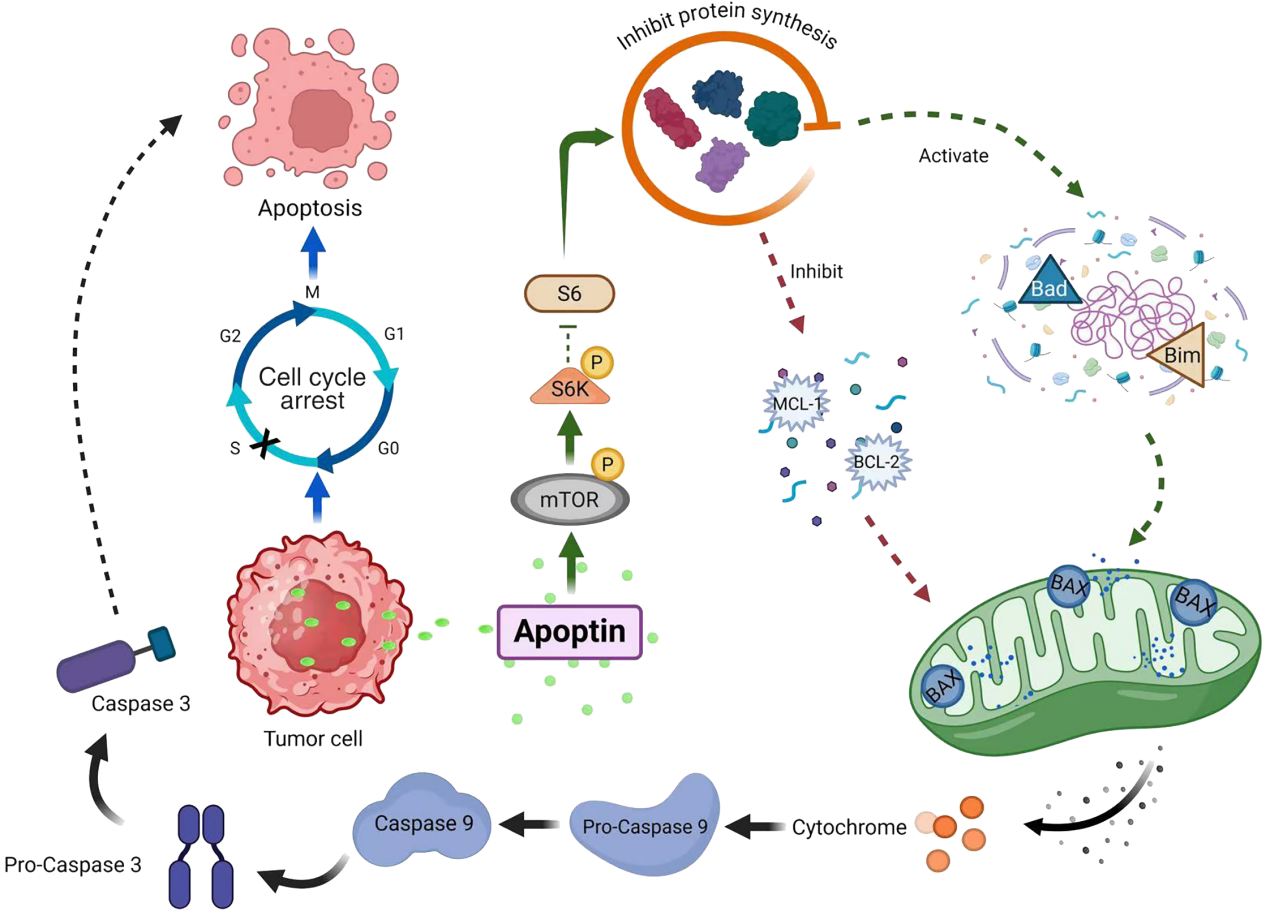
Ad-VT promotes the phosphorylation of mTOR/S6K signal, which leads to apoptosis of MCF-7 cells.

Our experiment confirmed that Ad-VT infection in MCF-7 cells leads to altered phosphorylation of mTOR and S6K proteins, suggesting potential involvement of the mTOR/S6K signalling pathway in apoptin-induced apoptosis (**Figure 8**). However, the exact molecular mechanisms remain to be fully elucidated. These preliminary findings, while indicative, are subject to certain limitations and require more comprehensive investigation to establish definitive mechanistic insights.

Living imaging technology is a new noninvasive technology for obtaining biomedical images of living tissue at the cellular and molecular levels and can be used for continuous monitoring of disease *in vivo*. Compared with other technologies, *in vivo* imaging is noninvasive, highly sensitive and allows dynamic monitoring, which is advantageous for the quantitative evaluation of cell proliferation and tumours in the body.

In this study, the firefly luciferase-labelled human breast cancer cell line McF-7-luc, which has the highest luciferase activity and good stability, was selected for comparison with MCF-7 cells *in vitro*. The biological characteristics of the constructed firefly luciferase-labelled McF-7-luc and MCF-7 cells were not significantly different, and a subcutaneous tumour-bearing model of McF-7-luc cells in nude mice was subsequently constructed. *In vivo* imaging revealed that the average bioluminescence intensity of tumours in the Ad-VP3 treatment group and Ad-VT treatment group was always lower than that in the other treatment groups after treatment and gradually decreased. In the control group, saline group and Ad-MOCK groups, the average tumour bioluminescence intensity was increased. Tumour growth curves revealed that the Ad-VP3 treatment and Ad-VT treatment inhibited tumour growth, whereas the other three treatments did not. Survival curves of nude mice revealed that the Ad-VP3 treatment group and the Ad-VT treatment group exhibited obviously prolonged survival to a significantly greater extent than the control group. The recombinant adenoviruses carrying apoptin, namely, Ad-VP3 and Ad–VT, slowed the tumour growth rate and extended the survival of the mice.

In conclusion, Apoptin can significantly inhibit the growth of MCF-7 human breast cancer cells, mainly by inducing the apoptotic death of MCF-7 cells. Proteomic experiments revealed that Ad-VT promoted the apoptosis of MCF-7 cells via the upregulation of the phosphorylated mTOR and S6K proteins. These findings suggest that Ad-VT can significantly enhance the apoptosis level of breast cancer cells, which is induced by the mTOR/S6K signalling pathway (**Figure 8**). *In vivo* experiments revealed that the recombinant adenovirus Ad-VT effectively inhibited the growth of tumour cells and prolonged the survival of the mice. This discovery provides a new idea and method for the treatment of breast cancer, and also provides a possibility for individualised combined therapy.

## Data Availability

The original contributions presented in the study are included in the article/**Supplementary Material**. Further inquiries can bedirected to the corresponding authors.

## References

[1] Ghafouri-Fard S Khanbabapour Sasi A Abak A Shoorei H Khoshkar A Taheri M. Contribution of miRNAs in the pathogenesis of breast cancer. Front Oncol. (2021) 11:768949. doi: 10.3389/fonc.2021.768949 34804971 PMC8602198

[2] Jungles KM Holcomb EA Pearson AN Jungles KR Bishop CR Pierce LJ. Updates in combined approaches of radiotherapy and immune checkpoint inhibitors for the treatment of breast cancer. Front Oncol. (2022) 12:1022542. doi: 10.3389/fonc.2022.1022542 36387071 PMC9643771

[3] Liang Y Wang Y Zhang Y Ye F Luo D Li Y. HSPB1 facilitates chemoresistance through inhibiting ferroptotic cancer cell death and regulating NF-κB signaling pathway in breast cancer. Cell Death Dis. (2023) 14:434. doi: 10.1038/s41419-023-05972-0 37454220 PMC10349816

[4] Cho Y-W Kim E-J Nyiramana MM Shin EJ Jin H Ryu JH. Paroxetine Induces Apoptosis of Human Breast Cancer MCF-7 Cells through Ca2+-and p38 MAP Kinase-Dependent ROS Generation. Cancers. (2019) 11:64. doi: 10.3390/cancers11010064 30634506 PMC6356564

[5] Shao W Wang X Liu Z Song X Wang F Liu X. Cyperotundone combined with adriamycin induces apoptosis in MCF-7 and MCF-7/ADR cancer cells by ROS generation and NRF2/ARE signaling pathway. Sci Rep. (2023) 13:1384. doi: 10.1038/s41598-022-26767-x 36697441 PMC9877033

[6] Tohkayomatee R Reabroi S Tungmunnithum D Parichatikanond W Pinthong D. Andrographolide Exhibits Anticancer Activity against Breast Cancer Cells (MCF-7 and MDA-MB-231 Cells) through Suppressing Cell Proliferation and Inducing Cell Apoptosis via Inactivation of ER-α Receptor and PI3K/AKT/mTOR Signaling. Molecules. (2022) 27:3544. doi: 10.3390/molecules27113544 35684480 PMC9182433

[7] Xue K-H Jiang Y-F Bai J-Y Zhang DZ Chen YH Ma JB. Melatonin suppresses Akt/mTOR/S6K activity, induces cell apoptosis, and synergistically inhibits cell growth with sunitinib in renal carcinoma cells via reversing Warburg effect. Redox Rep. (2023) 28:2251234. doi: 10.1080/13510002.2023.2251234 37642220 PMC10472857

[8] Zeng H Fu R Yan L Huang J. Lycorine induces apoptosis of A549 cells via AMPK-mammalian target of rapamycin (mTOR)-S6K signaling pathway. Med Sci Monitor. (2017) 23:2035–41. doi: 10.12659/msm.900742 PMC5421746 28450693

[9] Feng C Liang Y Teodoro JG. The role of apoptin in chicken anemia virus replication. Pathogens. (2020) 9:294. doi: 10.3390/pathogens9040294 32316372 PMC7238243

[10] Poon IKH Oro C Dias MM Zhang JP Jans DA. A tumor cell-specific nuclear targeting signal within chicken anemia virus VP3/apoptin. J virology. (2005) 79:1339–41. doi: 10.1128/JVI.79.2.1339-1341.2005 PMC538590 15613363

[11] Wyatt J Chan YK Hess M Tavassoli M Müller MM. Semisynthesis reveals apoptin as a tumour-selective protein prodrug that causes cytoskeletal collapse. Chem Science. (2023) 14:3881–92. doi: 10.1039/d2sc04481a PMC10074440 37035694

[12] Brown AC Reddy V Lee J Nair V. Marek’s disease virus oncoprotein Meq physically interacts with the chicken infectious anemia virus-encoded apoptotic protein apoptin. Oncotarget. (2018) 9:28910–20. doi: 10.18632/oncotarget.25628 PMC6034753 29988968

[13] Zhang GF Li CX Liu ZQ Ma S Chen HB. Cancer stem cell targets - a review. Eur Rev Med Pharmacol Sci. (2016) 20:2045–51. 27249603

[14] Rollano Peñaloza OM Lewandowska M Stetefeld J Ossysek K Madej M Bereta J. Apoptins: selective anticancer agents. Trends Mol medicine. (2014) 20:519–28. doi: 10.1016/j.molmed.2014.07.003 25164066

[15] Heilman DW Teodoro JG Green MR. Apoptin nucleocytoplasmic shuttling is required for cell type-specific localization, apoptosis, and recruitment of the anaphase-promoting complex/cyclosome to PML bodies. J virology. (2006) 80:7535–45. doi: 10.1128/JVI.02741-05 PMC1563728 16840333

[16] Los M Panigrahi S Rashedi I Mandal S Stetefeld J Essmann F. Apoptin, a tumor-selective killer. Biochim Biophys Acta. (2009) 1793:1335–42. doi: 10.1016/j.bbamcr.2009.04.002 19374922

[17] Du J Zhang Y Xu C Xu X. Apoptin-modified human mesenchymal stem cells inhibit growth of lung carcinoma in nude mice. Mol Med reports. (2015) 12:1023–9. doi: 10.3892/mmr.2015.3501 PMC4438975 25816208

[18] Birame BM Jigui W Fuxian Y Jiazeng S Zhili L Weiquan L. Potentiation of Apoptin-induced apoptosis by Cecropin B-like antibacterial peptide ABPs1 in human HeLa cervical cancer cell lines is associated with membrane pore formation and caspase-3 activation. J Microbiol Biotechnol. (2014) 24:756–64. doi: 10.4014/jmb.1209.09076 24633228

[19] Song G Fang J Shang C Li Y Zhu Y Xiu Z. Ad-apoptin inhibits glycolysis, migration and invasion in lung cancer cells targeting AMPK/mTOR signaling pathway. Exp Cell Res. (2021) 409:112926. doi: 10.1016/j.yexcr.2021.112926 34793774

[20] Zhou D Liu W Liang S Sun B Liu A Cui Z. Apoptin-derived peptide reverses cisplatin resistance in gastric cancer through the PI3K-AKT signaling pathway. Cancer Med. (2018) 7:1369–83. doi: 10.1002/cam4.2018.7.issue-4 PMC5911602 29522284

[21] Malla WA Arora R Khan RIN Mahajan S Tiwari AK. Apoptin as a tumor-specific therapeutic agent: current perspective on mechanism of action and delivery systems. . Front Cell Dev Biol. (2020) 8:524. doi: 10.3389/fcell.2020.00524 32671070 PMC7330108

[22] Li Y Zhu Y Fang J Li W Li S Liu X. Apoptin regulates apoptosis and autophagy by modulating reactive oxygen species (ROS) levels in human liver cancer cells. Front Oncol. (2020) 10:1026. doi: 10.3389/fonc.2020.01026 32714864 PMC7344208

[23] Liu Z Li Y Zhu Y Li N Li W Shang C. Apoptin induces pyroptosis of colorectal cancer cells via the GSDME-dependent pathway. Int J Biol Sci. (2022) 18:717–30. doi: 10.7150/ijbs.64350 PMC8741846 35002520

[24] Dalidowska I Gazi O Sulejczak D Przybylski M Bieganowski P. Heat shock protein 90 chaperones E1A early protein of adenovirus 5 and is essential for replication of the virus. Int J Mol Sci. (2021) 22(4):2020. doi: 10.3390/ijms22042020 33670684 PMC7921956

[25] Mou J Li C Zheng Q Meng X Tang H. Research progress in tumor angiogenesis and drug resistance in breast cancer. Cancer Biol Medicine. (20249), 1–15. doi: 10.20892/j.issn.2095-3941.2023.0515 PMC11271221 38940663

[26] Al Wafai R El-Rabih W Katerji M Safi R El Sabban M El-Rifai O. Chemosensitivity of MCF-7 cells to eugenol: release of cytochrome-c and lactate dehydrogenase. Sci Rep. (2017) 7:43730. doi: 10.1038/srep43730 28272477 PMC5341120

[27] Ciccone V Terzuoli E Donnini S Giachetti A Morbidelli L Ziche M. Stemness marker ALDH1A1 promotes tumor angiogenesis via retinoic acid/HIF-1α/VEGF signalling in MCF-7 breast cancer cells. J Exp Clin Cancer Res. (2018) 37:311. doi: 10.1186/s13046-018-0975-0 30541574 PMC6291966

[28] Kumar A Alasmari A. Achillea fragrantissima (Forssk.) Sch.Bip instigates the ROS/FADD/c-PARP expression that triggers apoptosis in breast cancer cell (MCF-7). PloS One. (2024) 19:e0304072. doi: 10.1371/journal.pone.0304072 38820323 PMC11142488

[29] Paramanantham A Kim MJ Jung EJ Kim HJ Chang SH Jung JM. Anthocyanins Isolated from Vitis coignetiae Pulliat Enhances Cisplatin Sensitivity in MCF-7 Human Breast Cancer Cells through Inhibition of Akt and NF-κB Activation. Molecules. (2020) 25:3623. doi: 10.3390/molecules25163623 32784919 PMC7466154

[30] Doronin K Kuppuswamy M Toth K Tollefson AE Krajcsi P Krougliak V. Tissue-specific, tumor-selective, replication-competent adenovirus vector for cancer gene therapy. J virology. (2001) 75:3314–24. doi: 10.1128/JVI.75.7.3314-3324.2001 PMC114124 11238857

[31] Gao J Zhang W Ehrhardt A. Expanding the spectrum of adenoviral vectors for cancer therapy. Cancers. (2020) 12:1139. doi: 10.3390/cancers12051139 32370135 PMC7281331

[32] Kaufman HL Kohlhapp FJ Zloza A. Oncolytic viruses: a new class of immunotherapy drugs. Nat Rev Drug Discovery. (2015) 14:642–62. doi: 10.1038/nrd.2016.178 PMC7097180 26323545

[33] Malogolovkin A Gasanov N Egorov A Weener M Ivanov R Karabelsky A. Combinatorial approaches for cancer treatment using oncolytic viruses: projecting the perspectives through clinical trials outcomes. Viruses. (2021) 13:1271. doi: 10.3390/v13071271 34209981 PMC8309967

[34] Bae Y Song SJ Mun JY Ko KS Han J Choi JS. Apoptin gene delivery by the functionalized polyamidoamine (PAMAM) dendrimer modified with ornithine induces cell death of hepG2 cells. Polymers. (2017) 9:197. doi: 10.3390/polym9060197 30970874 PMC6432117

[35] Burek M Maddika S Burek CJ Daniel PT Schulze-Osthoff K Los M. Apoptin-induced cell death is modulated by Bcl-2 family members and is Apaf-1 dependent. Oncogene. (2005) 25:2213–22. doi: 10.1038/sj.onc.1209258.P PMC2954965 16288204

[36] Chaabane W Cieślar-Pobuda A El-Gazzah M Jain MV Rzeszowska-Wolny J Rafat M. Human-gyrovirus-apoptin triggers mitochondrial death pathway—Nur77 is required for apoptosis triggering. Neoplasia. (2014) 16:679–93. doi: 10.1016/j.neo.2014.08.001 PMC4234882 25246270

[37] Guan G-f Zhao M Liu L-m Jin CS Sun K Zhang DJ. Salmonella typhimurium mediated delivery of apoptin in human laryngeal cancer. Int J Med Sci. (2013) 10:1639–48. doi: 10.7150/ijms.6960 PMC3805922 24155656

[38] Li JUN Wang H Ma Z Fan W Li Y Han B. TAT-Apoptin induces apoptosis in the human bladder cancer EJ cell line and regulates Bax, Bcl-2, caspase-3 and survivin expression. Exp Ther Medicine. (2012) 3:1033–8. doi: 10.3892/etm.2012.520 PMC3438815 22970013

[39] Tavassoli M Guelen L Luxon BA Gäken J. Apoptin: Specific killer of tumor cells? Apoptosis. (2005) 10:717–24. doi: 10.1007/s10495-005-0930-3 PMC3533135 16133863

[40] Zhou D Liu W Liang S Sun B Liu A Cui Z. Apoptin-poptinu peptide reverses cisplatin resistance in gastric cancer through the PI3K–AKT signaling pathway. Cancer Medicine. (2018) 7:1369–83. doi: 10.1002/cam4.1380 PMC5911602 29522284

