# Supplementary material for: Effect of bispecific recombinant oncolytic adenovirus carrying apoptin on apoptosis of MCF-7 cells
Source: Front Immunol. 2025 May 16;16:1530583. doi: 10.3389/fimmu.2025.1530583 (PMC12122747; doi:10.3389/fimmu.2025.1530583)
Supplement: Supplementary file 1 [file DataSheet1.pdf]

1. Construction of recombinant oncolytic adenovirus

(1)Strains, cells and plasmids

Escherichia coli competent bacteria JM109; packaging cell EETK-293, Plasmid pHAXI-Apoptin, pKS-PlolyA-hTERTp-E1a, pMT-Apoptin and pAd5 were both preserved by this experiment.

(2)Reagents

Fetal bovine serum, DMEM, and optiMEM culture medium were purchased from Gibco; SpeI, EcoRI and other restriction enzymes were purchased from NEB; DL2000 DNA Marker, dNTP, Ex-Taq DNA polymerase, etc. were purchased from Dalian Bao Bioengineering Company. (3)Construction of shuttle carrier pAd-VT

The plasmid pacAd5CMEVK-NpA was cleaved by indIII enzyme. After filling, the plasmid pacAd5CMEVK-NpA was cleaved with EcoRII and was ligated with the Apoptin gene fragment obtained after EcoRII/EcoRII double enzyme digestion, and the plasmid pAd-Apoptin was constructed. BamH I enzyme was used to cleave pAd Apoptin, which was then leveled and digested with Spe I enzyme to recover the linearized product; XhoI enzyme digestion of pKS-PolyA-hTTEER-E1a. After filling, slicing with SPEIenzyme was obtained to obtain fragments containing -PolyA-hTTEER-E1a, and link them to linearized pAd-Apoptin to construct a shuttle vector pAd-Apoptin-PolyA-hTERT-p-E1a, named pAd-VT.

2. Determination of caspase-3 activity

MCF-7 monolay cells were prepared from 6-well plates with a cell density of 1×10^6 cells/well. After being cultured at 37°C and 5% CO_2 for 24 h, MCF-7 cells were infected with 100 MOI each by recombinant adenovirus Ad-MOCK, Ad-VT and Ad-VP3 carrying apoptin for 24, 48h and 72h. Uninfected cells were used as control group at the same time.After reaching the action time, cells were collected in cell lysate, and centrifuged at 10000×g and 4°C for 1min after 10min in ice bath. Protein quantification of supernatant was performed using nucleic acid protein detector, and activity of supernatant containing 100μg protein was detected at 405nm using caspase-3 detection kit.

Ad-MOCK, Ad-VT and Ad-VP3 treated cells were treated for 24h, 48h and 72h and caspase-3 activity was detected by specific hair color substrate, that is, A405 value was compared with blank control group and Ad-MOCK group(fig.1). The activity of caspase-3 in the Ad-VT treatment group was significantly increased after 72 hours ($P < 0{\cdot}05$), indicating that Ad-VT could enhance the activity of caspase-3.

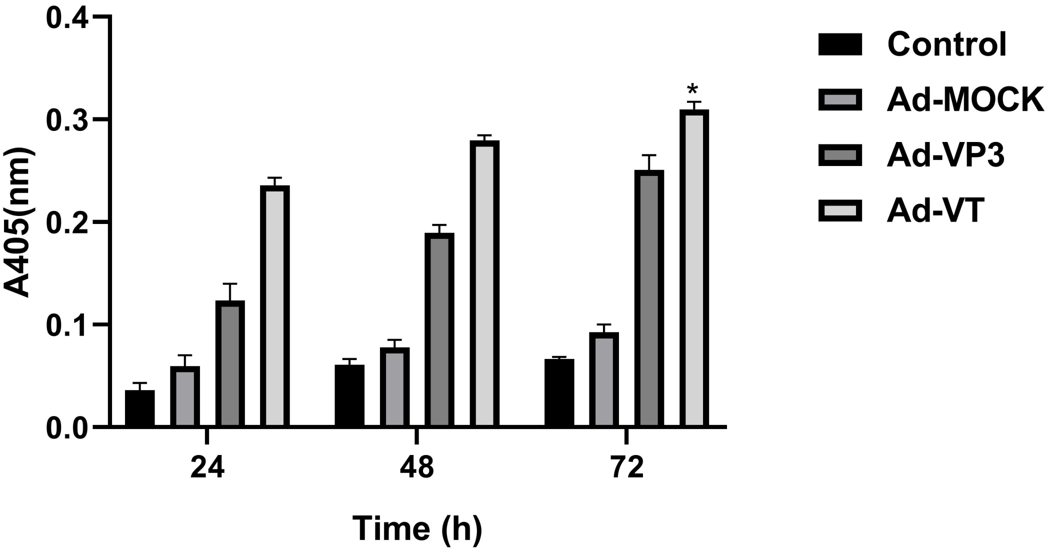

FIG.1 Effect of recombinant oncolytic adenovirus on Caspase 3 activity in MCF-7 cells. All the measurements were performed in triplicate, and the means ± standard deviations were compared with those of the control group (*P<0.05, **P<0.01, and ***P<0.001)

3. FIG.2 Ad-VT regulates apoptosis of breast cancer cells through mTOR/S6K signaling pathway

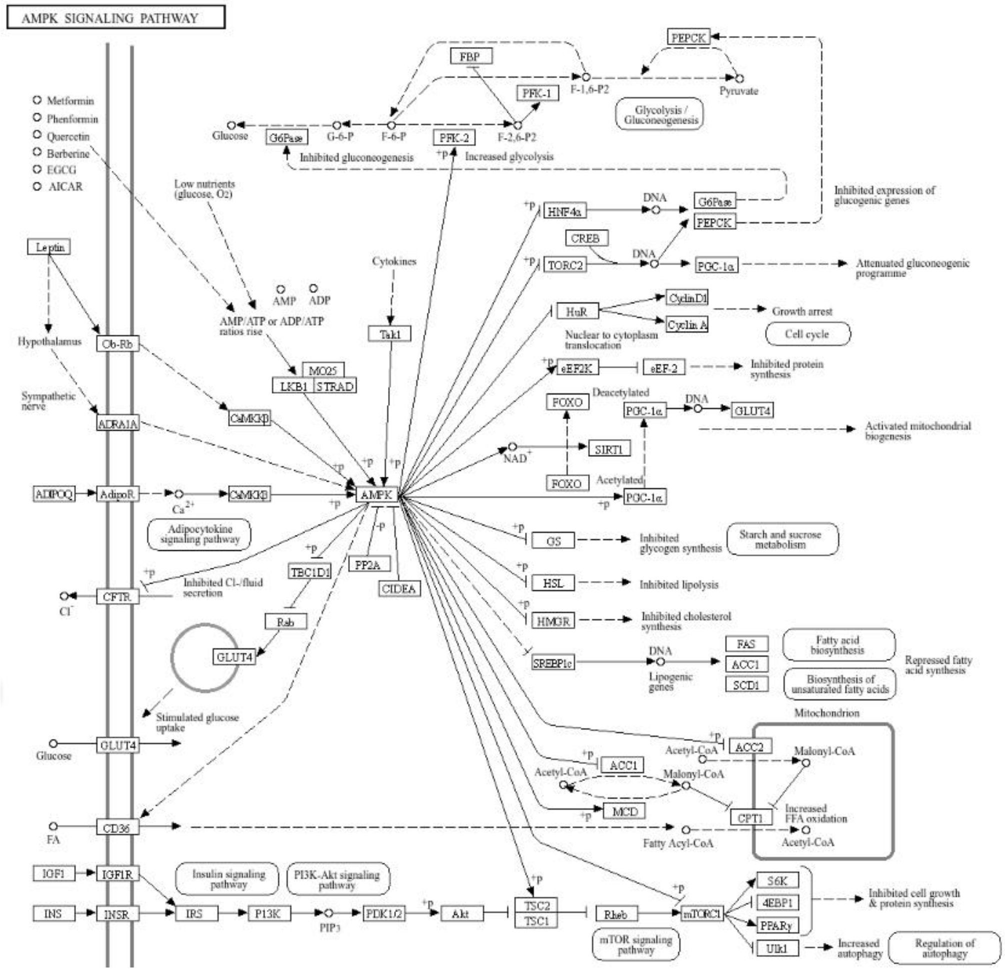

4. Effect of Apoptin on breast cancer cells MCF-7 cells

Cell culture and plasmid transfection. MCF-7 cells were transfected with 10 μ g recombinant plasmid pVAX1-Apoptin and empty plasmid pVAX1 (plasmid stored in our laboratory) by liposome mediated method, and blank cells were set up as control cells.

Materials and methods

(1)Cell killing rate was measured by MTS. MCF-7 cells transfected with plasmids were cultured for 24, 48 and 72h, and one 96-well cell culture plate was removed at three different time points (24h, 48h and 72h), the culture medium was discarded, 110 μ L was added to each well of MTS (1:10 dilution), and the cells were incubated for 80min in a warm tank and protected from light.The absorbance of the MCF-7 cells in each well was measured with an enzyme labelling instrument (Tecan Trading AG, Switzerland) at a wavelength of 490 nm for 20 s, and the cell activity inhibition rates of three recombinant oncolytic adenoviruses and control were calculated. Cell proliferation inhibition rate (%) = (absorbance value of control well-absorbance value of treatment well)/absorbance value of control well × 100%

(2)Apoptosis was detected by flow cytometry. Plasmid transfected MCF-7 cells were cultured for 24, 48 and 72h and treated with fitc Annexin-V apoptosis kit

(Becton Dickinson). The negative control group, Annexin V-FITC single staining group and PI single staining group were established. During computer processing, samples were examined using FACSCalibur flow cytometry, which was completed within 1 hour. Apoptosis was detected by flow cytometry (C6 Plus and FACSCalibur, Becton Dickinson, Franklin Lakes, NJ, USA)

Result

Inhibition of proliferation of MCF-7 cells by pVAX1-Apoptin

Recombinant plasmid pVAX1-Apoptin significantly inhibited the proliferation of MCF-7 cells (P < 0·01) with a inhibition rate of 22% after transfection for 48h (fig. 3).Due to the cytotoxic effect of liposomes, pVAX1 transfection group showed a certain killing effect after transfection, but then the cells gradually resumed vigorous growth.

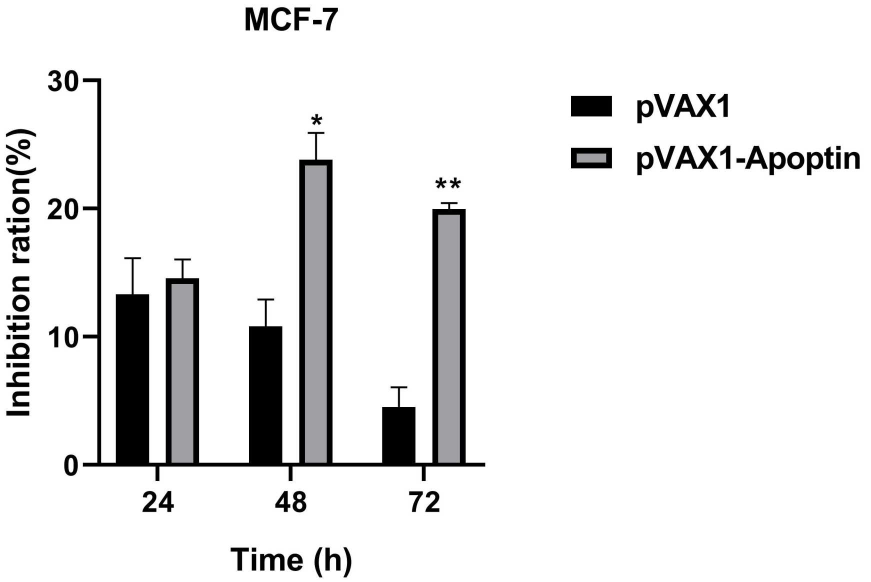

FIG.3 Inhibition of MCF-7 cell proliferation by pVAX1-Apoptin.All the measurements were performed in triplicate, and the means ± standard deviations were compared with those of the pVAX1 group (*P<0.05, **P<0.01, and ***P<0.001)

Effect of pVAX1-Apoptin on apoptosis of MCF-7 cells

After transfection with recombinant plasmid pVAX1-Apoptin for 48h, the apoptosis level of MCF-7 cells increased, and the apoptosis rate could reach more than 20% (fig. 4).

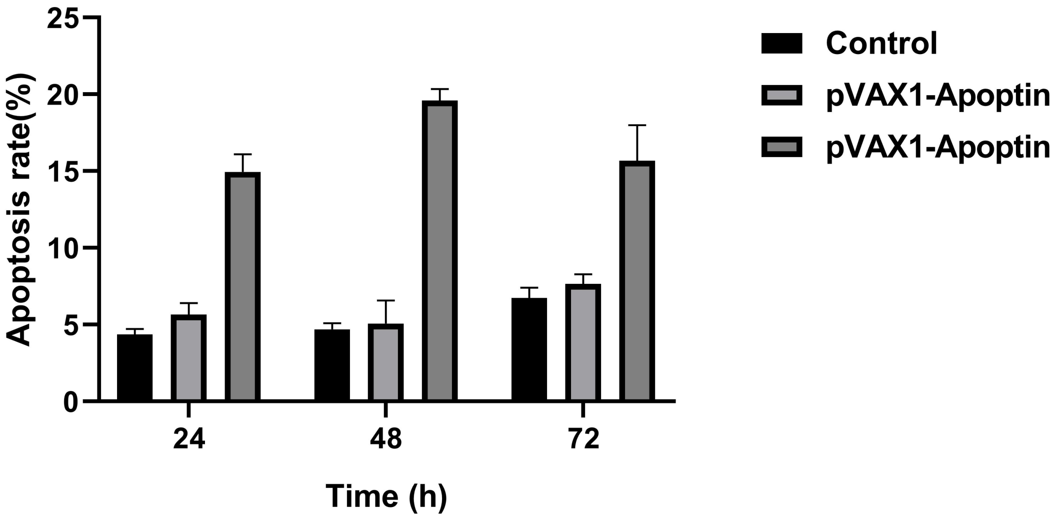

FIG.4 Apoptosis of MCF-7 cells by pVAX1-Apoptin. All the measurements were performed in triplicate, and the means ± standard deviations were compared with those of the control group (*P<0.05, **P<0.01, and ***P<0.001)
