# Supplementary material for: Effect of bispecific recombinant oncolytic adenovirus carrying apoptin on apoptosis of MCF-7 cells
Source: Front Immunol. 2025 May 16;16:1530583. doi: 10.3389/fimmu.2025.1530583 (PMC12122747; doi:10.3389/fimmu.2025.1530583)

1. Differentially expressed protein

**Protein IDs**	**pathway ID**	**Uniprot ID**	**Ensembl Gene ID**	**Description**	**48CON**	**48VT**	**Protein_Ratio（48VT/48CON）**	**Protein_Status（48VT/48CON）**
M3K1_HUMAN	hsa:4214	Q13233	ENSG00000095015	mitogen-activated protein kinase kinase kinase 1, E3 ubiquitin protein ligase [Source:HGNC Symbol;Acc:HGNC:6848]	54.2164	76.25587	1.406509	UP
S6K_HUMAN	hsa:6198	P23443	ENSG00000108443	ribosomal protein S6 kinase, 70kDa, polypeptide 1 [Source:HGNC Symbol;Acc:HGNC:10436]	200.1661	266.1314	1.329552	UP
MYC_HUMAN	hsa:23077	O75592	ENSG00000005810	MYC binding protein 2, E3 ubiquitin protein ligase [Source:HGNC Symbol;Acc:HGNC:23386]	1980.839	2587.304	1.306166	UP
STK3_HUMAN	hsa:6788	Q13188	ENSG00000104375	serine/threonine kinase 3 [Source:HGNC Symbol;Acc:HGNC:11406]	3927.289	4782.631	1.217794	UP
KS6A6_HUMAN	hsa:27330	Q9UK32	ENSG00000072133	ribosomal protein S6 kinase, 90kDa, polypeptide 6 [Source:HGNC Symbol;Acc:HGNC:10435]	11889.63	13867.89	1.166385	UP
FLNA_HUMAN	hsa:2316	P21333	ENSG00000196924	filamin A, alpha [Source:HGNC Symbol;Acc:HGNC:3754]	734126.3	799877.4	1.089564	UP
GBG12_H	hsa:55	Q9U	ENSG0000	guanine	5769.	5933.	1.028403	UP

UMAN	970	BI6	0172380	nucleotide binding protein (G protein), gamma 12 [Source:HGNC Symbol;Acc:HGNC:19663]	17	03		
PA24A_HUMAN	hsa:5321	P47712	ENSG00000116711	phospholipase A2, group IVA (cytosolic, calcium-dependent) [Source:HGNC Symbol;Acc:HGNC:9035]	175.1734	194.378	1.109631	UP
RICTR_HUMAN	hsa:253260	Q6R327	ENSG00000164327	RPTOR independent companion of MTOR, complex 2 [Source:HGNC Symbol;Acc:HGNC:28611]	746.9537	880.7778	1.17916	UP
STRAA_HUMAN	hsa:92335	Q7RTN6	ENSG00000266173	STE20-related kinase adaptor alpha [Source:HGNC Symbol;Acc:HGNC:30172]	493.0469	572.8974	1.161953	UP
IF4B_HUMAN	hsa:1975	P23588	ENSG00000063046	eukaryotic translation initiation factor 4B [Source:HGNC Symbol;Acc:HGNC:3285]	29177.29	29354.12	1.006061	UP
TSC1_HUMAN	hsa:7248	Q92574	ENSG00000165699	tuberous sclerosis 1 [Source:HGNC Symbol;Acc:HGNC:12362]	341.3984	285.3435	0.835808	DOWN
RS6_HUMAN	hsa:6194	P62753	ENSG00000137154	ribosomal protein S6 [Source:HGNC Symbol;Acc:HG	122706.1	103745.6	0.845481	DOWN

				NC:10429]				
AKTS1_HUMAN	hsa:84335	Q96B36	ENSG00000204673	AKT1 substrate 1 (proline-rich) [Source:HGNC Symbol;Acc:HGNC:28426]	1000.071	844.5211	0.844461	DOWN
KS6A1_HUMAN	hsa:6195	Q15418	ENSG00000117676	ribosomal protein S6 kinase, 90kDa, polypeptide 1 [Source:HGNC Symbol;Acc:HGNC:10430]	2681.419	2594.135	0.967449	DOWN

2. Ad-VT promotes the phosphorylation of mTOR/S6K signal, which leads to apoptosis of MCF-7 cells.

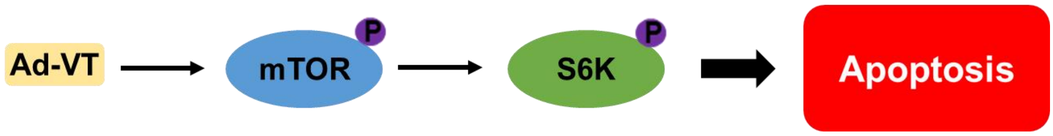

Supplement: Supplementary file 2 [file DataSheet2.pdf]
